# Neutralizing Antibodies Induced by First-Generation gp41-Stabilized HIV-1 Envelope Trimers and Nanoparticles

**DOI:** 10.1128/mBio.00429-21

**Published:** 2021-06-22

**Authors:** Sonu Kumar, Xiaohe Lin, Timothy Ngo, Benjamin Shapero, Cindy Sou, Joel D. Allen, Jeffrey Copps, Lei Zhang, Gabriel Ozorowski, Linling He, Max Crispin, Andrew B. Ward, Ian A. Wilson, Jiang Zhu

**Affiliations:** a Department of Integrative Structural and Computational Biology, The Scripps Research Institute, La Jolla, California, USA; b IAVI Neutralizing Antibody Center, The Scripps Research Institute, La Jolla, California, USA; c Consortium for HIV/AIDS Vaccine Development (CHAVD), The Scripps Research Institute, La Jolla, California, USA; d School of Biological Sciences, University of Southampton, Southampton, United Kingdom; e Skaggs Institute for Chemical Biology, The Scripps Research Institute, La Jolla, California, USA; f Department of Immunology and Microbiology, The Scripps Research Institute, La Jolla, California, USA; University of Pittsburgh School of Medicine

**Keywords:** envelope trimer, glycan holes, gp41 stabilization, HIV-1 vaccine design, nanoparticle, neutralizing antibodies

## Abstract

The immunogenicity of gp41-stabilized HIV-1 BG505 envelope (Env) trimers and nanoparticles (NPs) was recently assessed in mice and rabbits. Here, we combined Env-specific B-cell sorting and repertoire sequencing to identify neutralizing antibodies (NAbs) from immunized animals. A panel of mouse NAbs was isolated from mice immunized with a 60-meric I3-01 NP presenting 20 stabilized trimers. Three mouse NAbs potently neutralized BG505.T332N by recognizing a glycan epitope centered in the C3/V4 region on BG505 Env, as revealed by electron microscopy (EM), X-ray crystallography, and epitope mapping. A set of rabbit NAbs was isolated from rabbits immunized with a soluble trimer and a 24-meric ferritin NP presenting 8 trimers. Neutralization assays against BG505.T332N variants confirmed that potent rabbit NAbs targeted previously described glycan holes on BG505 Env and accounted for a significant portion of the autologous NAb response in both the trimer and ferritin NP groups. Last, we examined NAb responses that were induced by non-BG505 Env immunogens. We determined a 3.4-Å-resolution crystal structure for the clade C transmitted/founder (T/F) Du172.17 Env with a redesigned heptad repeat 1 (HR1) bend in gp41. This clade C Env, in a soluble trimer form and in a multivalent form with 8 trimers attached to ferritin NP, and the gp41-stabilized clade A Q482-d12 Env trimer elicited distinct NAb responses in rabbits, with notable differences in neutralization breadth. Although eliciting a broad NAb response remains a major challenge, our study provides valuable information on an HIV-1 vaccine design strategy that combines gp41 stabilization and NP display.

## INTRODUCTION

The envelope glycoprotein (Env) on HIV-1 virions mediates cell entry and is the target of broadly neutralizing antibodies (bNAbs) (1). Diverse bNAb families have been identified from HIV-1-infected individuals. The structural characterization of these human bNAbs in complex with Env proteins has defined multiple sites of HIV-1 vulnerability, including the CD4 binding site (CD4bs), quaternary V1/V2 glycan site, N332-oligomannose patch, silent face, gp120-gp41 interface, fusion peptide (FP), and membrane-proximal external region (MPER) (2–4). These human bNAbs often have unusual sequence characteristics that are acquired during extensive virus-host coevolution. As a result, targets of bNAbs differ substantially from strain-specific epitopes that are recognized by autologous NAbs early in human infection (5–8). Information about both types of antibodies and tracing them back to their unmutated common ancestors (UCAs) and early intermediates are valuable for guiding the rational design of vaccine immunogens (9–11).

Soluble native-like Env trimers have emerged as a promising platform for HIV-1 vaccine design (12, 13). As the leading design platform, SOSIP trimers have been created and characterized for diverse HIV-1 strains and subtypes (14–17), followed by native flexibly linked (NFL) (18) and uncleaved prefusion optimized (UFO) trimers (19). High-resolution Env structures, determined by X-ray crystallography and cryo-electron microscopy (cryo-EM), have provided a rational basis for improving trimer design (20–23). Mutations intended to increase Env stability and immunogenicity have been extensively tested on the basis of SOSIP and NFL trimers (24–36), whereas the main focus in the development of UFO trimers has been to reduce Env metastability. The N terminus of heptad repeat 1 (HR1_N_, residues 547 to 569) was identified as a major cause of Env metastability, leading to the design of cleaved, HR1-redesigned trimers or UFO trimers with removal of the cleavage site between gp120 and gp41 (19). UFO-BG trimers were derived for diverse HIV-1 Envs by replacing wild-type gp41 with BG505 gp41 of the UFO design (37). All of these design variants can be referred to as “gp41-stabilized” Env trimers because the modifications are within gp41. To further enhance immune recognition, SOSIP, NFL, and UFO trimers have been displayed on nanoparticles (NPs) of a diverse chemical nature, such as protein, lipid, and iron oxide (IO) NPs (30, 37–44). Various animal models, including mice, rabbits, and nonhuman primates (NHPs), have been used to assess the immunogenicity of HIV-1 Env in both soluble and particulate forms. To date, the BG505 SOSIP trimer has failed to elicit a detectable tier 2 NAb response in wild-type mice but generated robust nonneutralizing binding titers toward a “neoepitope” at its base (45). However, a tier 2 NAb response was observed for a large protein NP, I3-01 60-mer, presenting 20 HR1-redesigned BG505 trimers (37). Using various vaccine design strategies, mouse antibodies have been elicited to the N332-glycan supersite (nonneutralizing) (46) and fusion peptide (weakly neutralizing but broad) (47). A germ line-targeting strategy proved to be successful in engineered mice with knock-in genes encoding bNAbs and their precursors (48–50). In contrast to challenges of eliciting NAb responses in mice, potent and sometimes broad tier 2 NAb responses were reported in recent vaccine studies, in which rabbits were immunized with diverse Env trimers and trimer-presenting NPs (17, 27–30, 35, 51–53). However, epitope mapping indicated that specific “glycan holes” on the HIV-1 Env dominated the autologous NAb response in rabbits (54–61). HIV-1 immunogens in trimeric and particulate forms have also been assessed in NHPs, in which they were found to elicit consistent autologous but sparse cross-subtype NAb responses (17, 42, 51, 52, 59, 62–65). The C3/465 epitope was identified as a major target of NAb responses that were induced by the BG505 SOSIP trimer in macaques (59, 66). Overall, the recognition of Env by mouse (and to some extent macaque) NAbs is much less understood relative to rabbit NAbs. Additionally, the effect of both HR1 and gp41 stabilization, which is the core of UFO trimer design (19, 37), on NAb elicitation and epitope targeting in wild-type animal models has not been as well characterized as SOSIP and NFL trimers.

We previously designed gp41-stabilized trimers and NPs and assessed their NAb responses in mice and rabbits (37). In this study, we characterized mouse and rabbit NAbs that were induced by these immunogens in greater detail. First, we identified tier 2 mouse NAbs that were elicited by a 60-meric I3-01 NP presenting 20 HR1-redesigned BG505 trimers. A potent NAb, M4H2K1, was identified by pairing representative heavy and light chains obtained from the next-generation sequencing (NGS) analysis of Env-specific splenic B cells. Two somatically related NAbs were also isolated by single B-cell sorting and antibody cloning. Negative-stain EM (nsEM) revealed that M4H2K1 recognized the C3/V4 region of the native-like BG505 Env. The crystal structure of M4H2K1 bound to a BG505 gp120 core at 4.3-Å resolution delineated key antibody interactions with the C2/C3/V4/V5 epitope, which were confirmed in TZM-bl neutralization assays against a panel of BG505.T332N mutant viruses. A less potent NAb, M1H2K1, from a different mouse (M1) was also identified, which likely targeted the same epitope. We then performed single B-cell sorting and NGS for one rabbit immunized with an HR1-redesigned BG505 trimer and another with a ferritin (FR) NP presenting this trimer (37). Three selected rabbit NAbs were tested against a panel of glycan hole variants of BG505.T332N and found to target glycan holes at 241/289 and 465. Further analyses of plasma neutralization confirmed that these glycan holes accounted for a large portion of the polyclonal antibody response, suggesting that FR display cannot broaden the rabbit NAb response induced by soluble BG505 Env. Last, we determined a 3.4-Å-resolution crystal structure of an HR1-redesigned trimer derived from the Env of a clade C transmitted/founder (T/F) virus, Du172.17. In rabbits, the Du172.17 trimer and FR NP induced low titers of heterologous but not autologous NAb responses, whereas the UFO-BG trimer of a clade A T/F Q842-d12 Env exhibited primarily a tier 1 NAb response. Our study thus confirmed that protein NPs presenting gp41-stabilized BG505 trimers can induce potent tier 2 NAbs in mice and rabbits, but the elicitation of a broad NAb response remains a challenge for HIV-1 vaccine design.

## RESULTS

### Mouse NAbs isolated by Env-specific B-cell sorting and antibody NGS.

**FIG 1:**
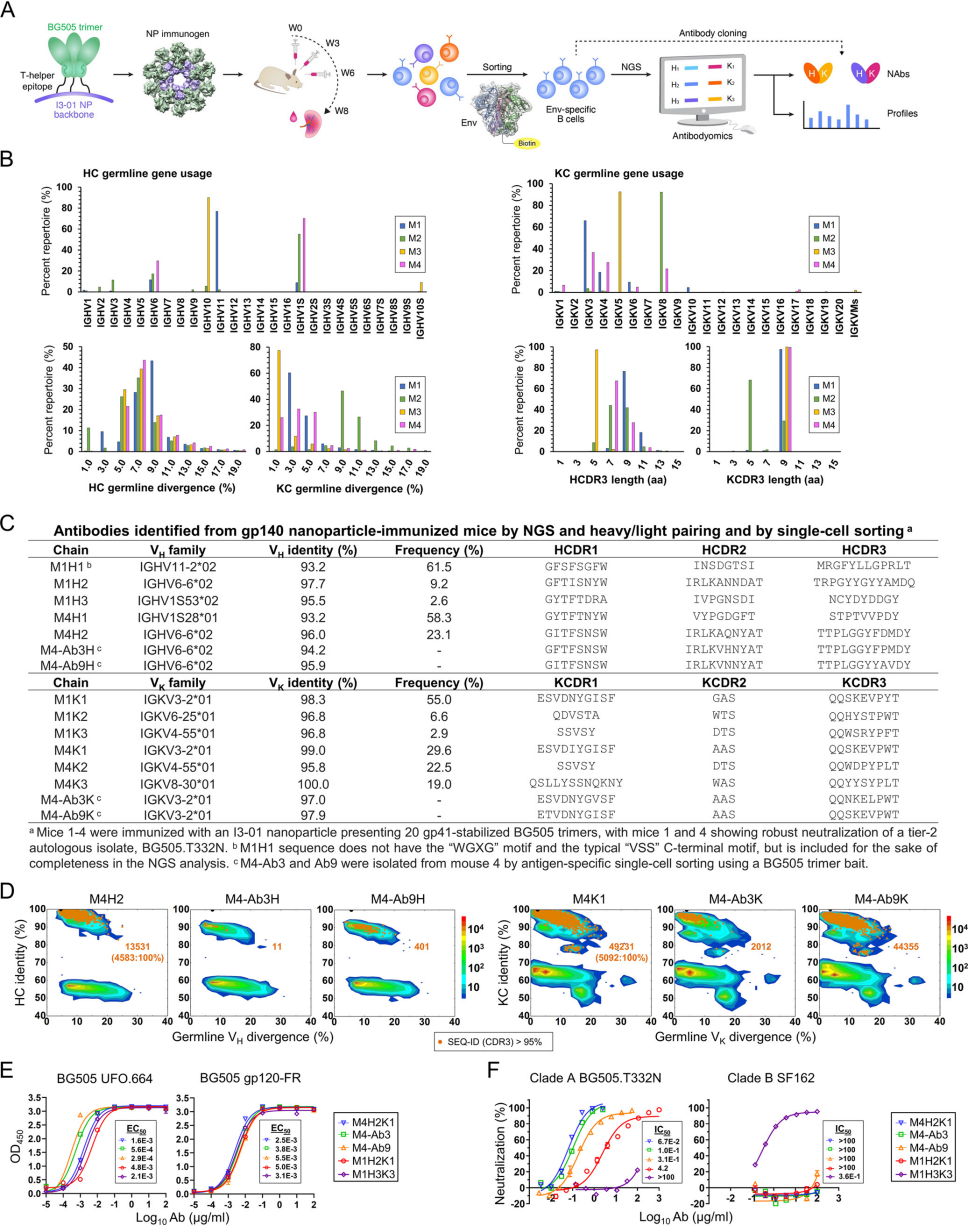
Tier 2 neutralizing antibodies isolated from gp140 nanoparticle-immunized mice. (A) Schematic representation of mouse immunization with BG505 gp140.664.R1-PADRE-I3-01 nanoparticle and antibody isolation from mouse splenic B cells using two approaches: Env-specific bulk B-cell sorting followed by next-generation sequencing (NGS) and Env-specific single B-cell sorting combined with antibody cloning. (B) Quantitative B-cell repertoire profiles derived from the NGS analysis of Env-specific splenic B cells from four mice in the I3-01 group, including germ line gene usage, the degree of somatic hypermutation (SHM), and CDR3 loop length. (C) Characteristics of antibody heavy and κ-light chains (HC and KC) identified from the clustering analysis of mouse NGS data and from single B-cell sorting and antibody cloning. (D) Divergence-identity analysis of murine NAbs in the context of Env-specific splenic B cells from mouse 4 (M4). HCs and KCs are plotted as a function of sequence identity to the template and sequence divergence from putative germ line genes. Color coding denotes sequence density. The template and sequences identified based on CDR3 identity of 95% or greater to the template are shown as black and orange dots on the plots, with the number of related sequences labeled accordingly. The number of sequences with 100% identity to the template is indicated in parentheses and labeled on the plot. (E) ELISA binding of mouse NAbs to the BG505 UFO.664 trimer and BG505 gp120-ferritin (FR) nanoparticle probe with EC_50_ values labeled next to the binding curves. (F) Percent neutralization of mouse NAbs against autologous tier 2 clade A BG505.TN332N and heterologous tier 1 clade B SF162 pseudoviruses with IC_50_ values labeled next to the neutralization curves. Five mouse NAbs including M4H2K1 (blue), M4-Ab3 (green), M4-Ab9 (orange), M1H2K1 (red), and M1H3K3 (purple) are shown in panels E and F.

We previously reported a tier 2 NAb response to mouse immunization with protein NPs presenting an HR1-redesigned BG505 trimer, termed gp140.664.R1 (37). A novel protein NP construct with 20 gp140.664.R1 trimers attached to the I3-01 60-mer with PADRE, a 13-amino-acid (aa) T-helper epitope (67), appeared to be most immunogenic. Here, we further isolated and characterized tier 2 NAbs from the I3-01 group, in which two mice (M1 and M4) developed a robust tier 2 NAb response after three injections. The HR1-redesigned BG505 trimer probe was utilized in two strategies to assist with NAb identification from mouse splenic B cells. One strategy focused on the NGS analysis of bulk-sorted B cells, and the other strategy involved single-cell sorting and antibody cloning (Fig. 1A). Notably, this trimer probe has been successfully used to identify early intermediates of the PGT121 lineage from a phage antibody library (68) and two N332-directed bNAbs from peripheral blood mononuclear cells (PBMCs) from an HIV-1-infected Chinese donor (69).

We first isolated mouse NAbs through the NGS analysis of bulk-sorted Env-specific B cells and random pairing of consensus heavy and light chains (Fig. 1A). This approach was based on the hypothesis that the small number of vaccine-induced B-cell lineages would enable the frequency-based identification of functional antibodies. In bulk sorting, 87 to 1,064 BG505 Env-specific B cells were obtained from the four mice studied (see Fig. S1A in the supplemental material). Unbiased mouse antibody heavy chain (HC) and κ-light chain (KC) libraries were constructed and sequenced on an Ion S5 platform, which yielded up to 1.22 million raw reads (Fig. S1B). The antibody NGS data were then processed using a mouse antibodyomics pipeline (70) to remove low-quality reads (Fig. S1B). Quantitative profiles of Env-specific B-cell populations were determined for each mouse in the I3-01 NP group, revealing distinct patterns (Fig. 1B). Diverse antibody variable (V_H_ and V_K_) genes were activated in response to Env immunization with some overlap for the two mice (M1 and M4) that developed a tier 2 autologous NAb response (37). IGHV6 and IGHV1S were used by Env-specific antibodies from both M1 and M4, in which 77% of M1 HCs were derived from IGHV11 and 70% of M4 HCs were derived from IGHV1S. The V_K_ distribution showed overlap of usage of the germ line genes IGKV3, IGKV4, and IGKV6. In terms of the degree of somatic hypermutation (SHM), a consistent V_H_ distribution was observed for four mice, which peaked at the 7 to 9% nucleotide (nt) difference from the assigned germ line genes. In contrast, four mice exhibited significant differences in their V_K_ SHM distributions, with M2 and M3 showing average SHMs of 10.0% and 1.6%, respectively. In terms of complementarity-determining region 3 (CDR3) length, M2 and M3 also appeared to exhibit a notable difference from M1 and M4 by using predominantly 5-aa KCDR3 and HCDR3 loops, respectively. Nonetheless, a CDR3-based clustering algorithm (68) was used to calculate consensus sequences for major HC and KC lineages from the M1 and M4 NGS data (Fig. 1C and Fig. S1C) because immunoglobulin G (IgG) purified from the sera of these two mice neutralized BG505.T332N (37). Interestingly, M1H2 and M4H2, both from the second largest sequence family, had an IGHV6-6*02 origin, whereas M1K1 and M4K1 shared the IGKV3-2*01 germ line gene (Fig. 1C). These consensus HCs and KCs were synthesized to reconstitute antibodies for functional validation. To further enrich the antibody pool, we performed single B-cell sorting on M4 splenic B cells using the same trimer probe (Fig. 1A). The natively paired HCs and KCs of two monoclonal antibodies (MAbs), M4-Ab3 and M4-Ab9, were derived from IGHV6-6*02 and IGKV3-2*01, suggesting that they might be somatically related to M4H2 and M4K1, respectively (Fig. 1C and Fig. S1D). Two-dimensional (2D) divergence/identity analysis (69, 71) was performed to compare the prevalence of these mouse MAbs in the NGS-derived antibody repertoire (Fig. 1D). Using an HCDR3 identity cutoff of 95%, 13,531, 11, and 401 sequences were related to M4H2, M4-Ab3 HC, and M4-Ab9 HC, respectively. Based on the same KCDR3 identity cutoff, 49,231, 2,012, and 44,355 sequences were somatically related to M4K1, M4-Ab3 KC, and M4-Ab9 KC, respectively. Notably, a significant portion of somatically related HCs and KCs were identical to M4H2 (33.9%) and M4K1 (10.3%), respectively, suggesting that these two consensus sequences represent native antibody chains that are used by Env-specific B cells from M4. Altogether, a panel of MAbs was identified from two NAb-producing mice in our previous study (37).

10.1128/mBio.00429-21.1 FIG S1 HIV-1 Env-specific sorting and NGS of mouse splenic B cells for antibody isolation. Download FIG S1, PDF file, 0.1 MB. Copyright © 2021 Kumar et al. 2021 Kumar et al. https://creativecommons.org/licenses/by/4.0/ This content is distributed under the terms of the Creative Commons Attribution 4.0 International license.

We further characterized the binding of these mouse MAbs to a panel of Env antigens by enzyme-linked immunosorbent assay (ELISA) (Fig. 1E and Fig. S2A). When the BG505 UFO.664 trimer was used as a coating antigen, three MAbs from M4, including NGS-derived M4H2K1 and single-cell-derived M4-Ab3 and M4-Ab9, and two NGS-derived MAbs from M1, M1H2K1 and M1H3K3, bound to this native-like Env trimer with up to a 16.6-fold difference in the half-maximal effective concentration (EC_50_) value (Fig. 1E, left). Among the three M4 MAbs, the two single-cell-derived MAbs bound to BG505 UFO.664 Env with 2.9- and 5.5-fold-lower EC_50_ values than M4H2K1. Other NGS-derived HC-KC pairs showed low or no trimer binding (Fig. S2A, top). We examined the epitope specificity of trimer-binding MAbs by testing four probes that displayed Env domains or epitopes on FR 24-mer, including a BG505 gp120-FR (41), an N332-FR termed 1GUT_A_ES-5GS-FR (70), a BG505 V1V2-FR (41), and an FP-5GS-FR. In ELISA, all five MAbs bound to BG505 gp120-FR with comparable EC_50_ values (Fig. 1E, right) but failed to show any detectable binding to the N332 supersite, V1V2 apex, or FP epitope in the context of the probes (Fig. S2A, bottom), suggesting that they may recognize a different epitope in gp120.

10.1128/mBio.00429-21.2 FIG S2 Functional evaluation of NGS and single-cell-derived mouse MAbs. Download FIG S2, PDF file, 0.8 MB. Copyright © 2021 Kumar et al. 2021 Kumar et al. https://creativecommons.org/licenses/by/4.0/ This content is distributed under the terms of the Creative Commons Attribution 4.0 International license.

We characterized the neutralizing activity of these mouse MAbs in TZM-bl assays (Fig. 1F; Fig. S2B and C). All trimer-binding MAbs, except M1H3K3, neutralized the autologous tier 2 BG505.T332N with up to a 63-fold difference in the half-maximal inhibitory concentration (IC_50_) value (Fig. 1F, left). The NGS-derived MAb from M4 (M4H2K1) appeared to be the most potent neutralizer, with an IC_50_ of 0.067 μg/ml, which was 2- to 5-fold higher than for bNAbs PGT121 (0.029 μg/ml) and PGT128 (0.013 μg/ml), respectively (69). In terms of potency, this mouse NAb was comparable to C3/V5-specific autologous NAbs that were isolated from NHPs, which showed a median IC_50_ of 0.06 μg/ml (66). Despite stronger Env binding, the single-cell-derived MAbs M4-Ab3 and M4-Ab9 neutralized BG505.T332N less effectively than the NGS-derived M4H2K1, with up to 4.6-fold-higher IC_50_ values. By comparison, other NGS-derived HC-KC pairs exhibited only low levels of autologous neutralization at high IgG concentrations (Fig. S2B, top). When tested against tier 1 clade B SF162, M1H3K3 yielded an IC_50_ of 0.36 μg/ml, whereas the other four autologous tier 2 NAbs did not exhibit any reactivity with SF162 (Fig. 1F, right; Fig. S2B, middle). None of the mouse MAbs neutralized the murine leukemia virus (MLV) Env-pseudotyped virus, except M4-Ab9, which showed some nonspecific signals at high IgG concentrations (Fig. S2B, bottom). Last, we assessed the neutralizing activity of these mouse MAbs against a global panel of 12 tier 2 isolates (72). Using MLV as a negative control in TZM-bl assays, M1H3K3 from M1 but not any of the M4 MAbs modestly neutralized two heterologous HIV-1 isolates, clade A/E pCNE8 and clade G pX1632 (Fig. S2C).

In brief, a panel of MAbs was identified from mice immunized with an I3-01 60-mer using a BG505 Env probe in two B-cell sorting strategies, followed by NGS and bioinformatics analyses. Pairing of the prevalent HCs and KCs identified by the NGS analysis of bulk-sorted Env-specific B cells provided an alternative to single-cell sorting, which captures MAbs in their native forms but can be technically challenging. As demonstrated here, these two strategies can be combined to identify and characterize vaccine-induced MAbs in the context of Env-specific B-cell repertoires. Functional evaluation confirmed that M4H2K1 was an autologous tier 2 NAb with high potency, whereas M1H3K3 was primarily a tier 1 NAb with some detectable heterologous tier 2 neutralizing activity. These two murine NAbs appear to target as-yet-unidentified epitopes in gp120.

### Autologous tier 2 mouse NAb M4H2K1 binds laterally to the BG505 Env trimer.

A modified BG505 SOSIP.664 trimer (RC1) on virus-like particles (VLPs) was previously shown to expand mouse germinal center (GC) B cells that were specific to the V3 glycan patch (46). In a recent study, Ringe et al. reported that mice immunized with SOSIP trimers attached to iron oxide (IO) NPs generated an autologous serum neutralizing response to the glycan hole at position 289 (44). Here, we combined nsEM and X-ray crystallography to elucidate the mechanism by which M4H2K1, one of the most potent murine NAbs identified to date, interacts with HIV-1 Env.

**FIG 2:**
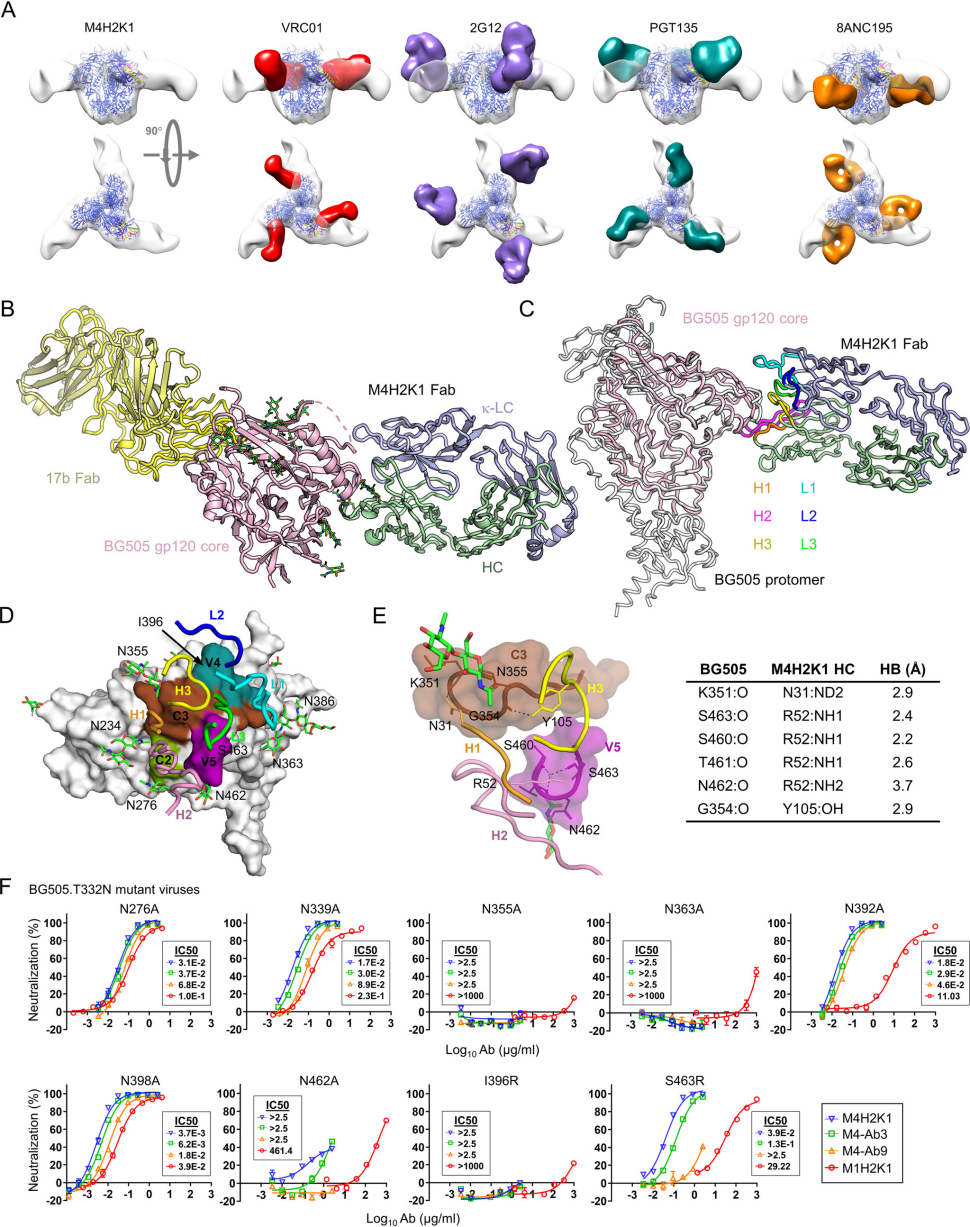
Structural epitope mapping of M4H2K1 on HIV-1 Env. (A) Three-dimensional (3D) EM reconstruction of M4H2K1 Fab/BG505 UFO.664 complex. The crystal structure of BG505 SOSIP.664 trimer (PDB ID: 4ZMJ) is docked into the EM trimer density and displayed in blue ribbons, with the potential M4H2K1 epitope color coded on the Env trimer structure (C3, yellow; V4, magenta; V5, green). Glycan moieties are shown as sticks. A closeup view of the epitope definition derived from the EM analysis can be found in Fig. S3A (left). Comparisons of the mode of M4H2K1 Fab binding to BG505 UFO.664 trimer with four bNAbs: VRC01 (red; EMD-6252), 2G12 (purple; EMD-5982), PGT135 (cyan; EMD-2331), and 8ANC195 (orange; EMD-2625). (B) Crystal structure of BG505 gp120 core (pink) in complex with Fabs 17b (yellow) and M4H2K1 at 4.3-Å resolution. (C) Side view of the crystal structure of the M4H2K1 Fab-BG505 gp120 core (light pink) complex superimposed onto one protomer of BG505 gp140 (gray) (PDB ID: 5CEZ). The M4H2K1 Fab is shown with the HCDR loops labeled and colored (H1, orange; H2, pink; H3, yellow) and LCDR loops (L1, cyan; L2, blue; L3, green). (D) Epitope of M4H2K1 Fab mapped onto the BG505 gp120 core shown in surface representation and defined as two residues containing an atom within 4.0 Å of each other (C2, green; C3, brown; V4, cyan; V5, pink). The M4H2K1 Fab is shown with HCDR and LCDR loops labeled and colored accordingly. (E) (Left) Hydrogen bonds are shown between HCDR loops (H3, yellow; H2, pink; H1, orange) and the gp120 core (C3, brown; V5, pink). (Right) Table listing residues involved in hydrogen-bond (HB) interaction with distances measured in Å. (F) Percent neutralization of mouse NAbs against nine BG505 mutant pseudoviruses with IC_50_ values labeled next to the neutralization curves. NAbs M4H2K1 (blue), M4-Ab3 (green), M4-Ab9 (orange), and M1H2K1 (red) are shown.

We first performed EM to visualize where the mouse NAb M4H2K1 binds on the BG505 Env trimer. We produced the antigen-binding fragment (Fab) of M4H2K1 and incubated it with the BG505 UFO.664 trimer to form a complex, which was then subjected to single-particle nsEM analysis (Fig. S3A). The three-dimensional (3D) reconstruction showed that the major species of this complex was Env trimers each bound to three M4H2K1 Fabs, with each Fab approaching the Env laterally (Fig. 2A, leftmost). After fitting a crystal structure of unliganded BG505 SOSIP.664 (PDB ID: 4ZMJ) (73) into the EM density, M4H2K1 was found to interact with an epitope that lies in the gp120 C3/V4 region. To determine the degree to which the M4H2K1 epitope overlaps neighboring bNAb epitopes, EM maps containing Fabs of four representative bNAbs, VRC01 (74), 2G12 (75), PGT135 (76), and 8ANC195 (77), were aligned to the M4H2K1 EM complex (Fig. 2A, right four structures). Our analysis revealed that M4H2K1 and VRC01 (but not other NAbs) would “clash” in their Env-bound mode as indicated by slightly overlapping EM densities, suggesting that the M4H2K1 epitope is in proximity to the CD4bs that is targeted by VRC01.

10.1128/mBio.00429-21.3 FIG S3 Structural characterization of the NGS-derived mouse NAb, M4H2K1. Download FIG S3, PDF file, 0.7 MB. Copyright © 2021 Kumar et al. 2021 Kumar et al. https://creativecommons.org/licenses/by/4.0/ This content is distributed under the terms of the Creative Commons Attribution 4.0 International license.

We then applied X-ray crystallography to further understand the molecular interactions of M4H2K1 with BG505 Env. To this end, we first obtained a crystal structure of M4H2K1 Fab at 1.50-Å resolution (Fig. S3B). In this structure, HCDR3 (10 aa) is sandwiched between HCDR1 and KCDR2, while KCDR3 (9 aa) fits between KCDR1 and HCDR2 (Fig. S3B). To gain more atomic details of the M4H2K1-Env interaction, we generated a gp120 core from the clade A BG505 Env to complex with Fab M4H2K1 and Fab 17b (78) to aid crystallization. A crystal structure of this complex was determined at 4.30-Å resolution in an orthorhombic (P2_1_2_1_2) crystal lattice. The structure showed that M4H2K1 Fab bound to the BG505 gp120 core by targeting the C2/C3/V4/V5 region (Fig. 2B and D). We then superimposed the M4H2K1 Fab-gp120 core complex onto a protomer of the BG505 SOSIP.664 trimer (PDB ID: 5CEZ) (79), which defined the orientation of M4H2K1 Fab HC and KC relative to BG505 Env in the lateral binding mode (Fig. 2C). The extended HCDR2 (19 aa) and KCDR1 (15 aa) engage the trimer in a pincer-like grasp (magenta and cyan loops in Fig. 2C). A total of 865 Å^2^ of the Fab is buried on the BG505 gp120 core surface, where HC and KC contribute to 70% and 30% of the Fab-buried surface area (BSA), respectively (Fig. 2D and Fig. S3C). The tip of the HCDR2 loop is deeply buried (348 Å^2^) inside the pocket formed by multiple parts of C2/C3/V5 (Fig. 2D and Fig. S3C) and makes most contact with Env, followed by KCDR1 with the next largest BSA of 193 Å^2^. Additionally, the other CDRs (BSA; H3, 117 Å^2^; H1, 130 Å^2^; L3, 67 Å^2^) and HC framework region 1 (BSA; HFR1, 9 Å^2^) are buried in the gp120 core surface except for KCDR2, which has no BSA (Fig. S3C). This analysis highlighted the importance of a long HCDR2 (19 aa) for anchoring M4H2K1 Fab to Env in a lateral orientation. Despite only moderate resolution, interactions at the interface of the BG505 gp120 core and M4H2K1 Fab were observed with little ambiguity. A hydrogen bond network at the interface appears to be formed by HCDR1 (N31), HCDR2 (R52), and HCDR3 (Y105) in M4H2K1 Fab and T278, R350, K351, N356, S460, T461, N462, and S463 in the BG505 gp120 core (Fig. 2E). To identify the regions involved in steric clashes between M4H2K1 and VRC01 Fabs upon EM fitting (Fig. 2A, panel 2 to the left), we superimposed the crystal structure of the M4H2K1 Fab-BG505 gp120 core complex onto the VRC01-bound BG505 F14 SOSIP trimer (PDB ID: 6V8X) (80). Overlap between the 19-aa-long HCDR2 loop of M4H2K1 Fab and LCDR1 of VRC01 Fab suggested competition between M4H2K1 and VRC01-class bNAbs for Env binding (Fig. S3D). Because of similar steric hindrance, IOMA-class bNAbs that target the CD4bs (81) could also compete with M4H2K1 for Env binding.

In a recent study, a group of NAbs was isolated from guinea pigs immunized with a BG505 SOSIP.664 trimer (82). Of these NAbs, CP506 Fab targeted the C3/V4 region of HIV-1 Env and neutralized BG505.T332N with an IC_50_ of 0.1 μg/ml. To compare the angles of approach between M4H2K1 and CP506 Fabs, we first constructed a model of the M4H2K1 Fab-bound BG505 Env trimer by superimposing our crystal structure of the M4H2K1 Fab-BG505 gp120 core complex onto the BG505 SOSIP.664 trimer (PDB ID: 4TVP) (83). We then docked this model into the 3D reconstruction of the CP506 Fab-BG505 SOSIP.664 trimer complex that was derived from the nsEM analysis (EMD-9003). A slight variation in the angle of approach was observed (Fig. S3E). Compared with CP506, which has a 16-aa HCDR2 and 8-aa HCDR3, M4H2K1 utilizes longer HCDR2 (19-aa) and HCDR3 (10-aa) to recognize HIV-1 Env. The crystal structure (Fig. 2B) suggests that glycans at N276, N339, N355, N363, and N462 and amino acids I396 and S463 (Fig. 2D and E) may be involved in BG505 Env recognition by M4H2K1. Glycans N234 and N386 point sideways with no direct contact with M4H2K1. To verify these interactions, we created nine BG505.T332N variants (N276A, N339A, N355A, N363A, N462A, I396R, and S463R, along with N392A and N398A, which are in proximity to the binding site) and tested their neutralization by four of the newly identified mouse NAbs (Fig. 2F). Glycan knockouts (KOs) at positions 276, 339, 392, and 398 and the S463R mutation exhibited only a negligible to modest effect, whereas glycan KOs at N355A, N363A, and N462A and the I396R mutation significantly reduced or completely abrogated neutralization by mouse NAbs (Fig. 2F). These findings suggest that glycans at N355 and N363, the contribution by a glycan positioned at N462, and I396 are critical for the M4H2K1-Env interaction. In contrast, glycans at N339, N363, and N392 were shown to be critical for Env recognition by CP506 (82). Last, we examined the conservation of three critical residues (N355, N363, and I396) in 6,966 HIV-1 Env sequences (www.hiv.lanl.gov/). A large proportion of isolates contain an NXT/S sequon at N355 (∼80%) and N462 (∼42%), similar to BG505, whereas positions 363 and 396 are less conserved in group M isolates (9% are Asn and 4.5% are Ile, respectively), leading to the autologous nature of NAb M4H2K1.

In summary, our structural analysis identified a critical epitope on BG505 Env that can be recognized by potent murine NAbs, and this was exemplified by M4H2K1. Compared with CP506, a guinea pig NAb that targets C3/V4 (82), M4H2K1 achieves higher potency by interacting with an expanded Env surface area that spans C2/C3/V4/V5. A less potent NAb, M1H2K1, was found from another mouse and exhibited similar sensitivity to a panel of BG505.T332N variants in the TZM-bl neutralization assays (Fig. 2F), suggesting that this NAb may recognize a similar epitope to M4H2K1, albeit with differential effects of mutations at N363, N392, and S463. Another NAb from M1, M1H3K3, neutralized heterologous strains but not autologous BG505.T332N. Because of the lack of structural data, we cannot exclude the possibility that the cross-neutralizing activity of M1H3K3 was caused by the random pairing of HCs and KCs. Therefore, the NP vaccine-induced mouse bNAbs may warrant further investigation in future studies.

### Stabilized BG505 trimer and FR NP elicit glycan hole NAbs in rabbits.

**FIG 3:**
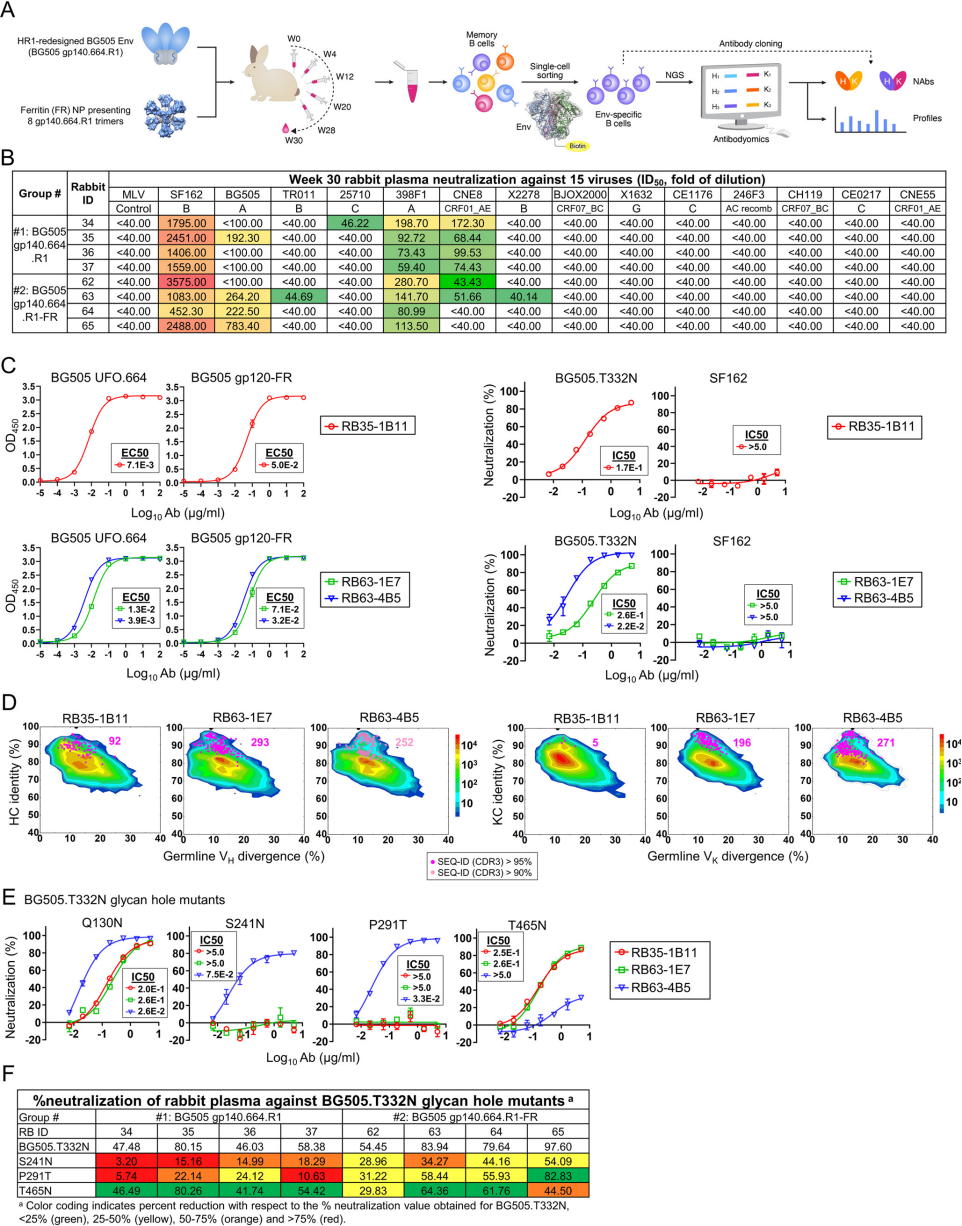
Tier 2 neutralizing antibodies isolated from rabbits immunized with HR1-redesigned BG505 gp140 trimer and its ferritin nanoparticle. (A) Schematic representation of rabbit immunization with the BG505-gp140.664.R1 trimer and ferritin (FR) nanoparticle and antibody isolation from rabbit PBMCs by Env-specific single B-cell sorting coupled with antibody cloning. (B) Neutralization (measured by ID_50_ values) of week 30 plasma from two rabbit groups against autologous tier 2 clade A BG505.T332N, tier 1 clade B SF162, and a 12-virus global panel, with MLV included as a negative control. Color coding indicates neutralization potency (red, potent; green, neutralizing but not potent; no color, nonneutralizing). (C) (Left) ELISA binding of rabbit NAbs to the BG505 UFO.664 trimer and BG505 gp120-FR NP probe with EC_50_ values labeled next to the ELISA curves. (Right) Percent neutralization of rabbit NAbs against autologous tier 2 clade A BG505.TN332N and heterologous tier 1 clade B SF162 pseudoviruses with IC_50_ values labeled next to the neutralization curves. Antibodies were diluted to 10 μg/ml and subjected to a 3-fold serial dilution in the TZM-bl assay. (D) Divergence-identity analysis of rabbit MAbs in the context of Env-specific rabbit B-cell repertoires from rabbits RB35 and RB63. Heavy and κ-light chain (HC and KC) sequences are plotted as a function of sequence identity to the template and sequence divergence from putative germ line genes. Color coding denotes sequence density. Templates and sequences identified based on the CDR3 identity cutoffs of 95% and 90% are shown as pink and light pink dots, respectively, on the plots with the number of sequences labeled accordingly. (E) Percent neutralization of rabbit NAbs against four BG505 pseudoviruses containing glycan hole mutations with IC_50_ values labeled next to the neutralization curves. NAbs RB35-1B11 (red), RB63-1E7 (green), and RB63-4B5 (blue) are shown in panels C and E. (F) Percent neutralization of rabbit plasma against BG505.T332N and its three glycan hole mutants. Color coding in the table indicates the percent reduction relative to the percent neutralization for BG505.T332N (<25%, green; 25 to 50%, yellow; 50 to 75%, orange; >75%, red).

Extensive studies of native-like trimers, particularly with the BG505 backbone, have revealed that the autologous NAb response in rabbits is mainly directed to “glycan holes” (54–61). Strategies that were intended to broaden the autologous NAb response in rabbit immunization with mixed SOSIP.664 trimers of two different clades and with the immune complex of a BG505 SOSIP.664 trimer and glycan hole MAb proved ineffective (84, 85). We previously immunized two groups of rabbits with an HR1-redesigned BG505 trimer (gp140.664.R1) and an FR NP displaying eight copies of this trimer (37). The gp140.664.R1-FR NP elicited a more rapid autologous tier 2 NAb response than the soluble trimer. Here, we isolated NAbs from these rabbits by combining single-cell sorting and NGS (Fig. 3A), similar to the mouse analysis (Fig. 1A). The characterization of these NAbs may provide additional insights into the effects of NP display, albeit using the smaller 24-meric FR NP, on the NAb response induced by Env immunization in rabbits.

We first assessed rabbit plasma at the last time point (week 30) against autologous tier 2 clade A BG505.T332N, tier 1 clade B SF162, and a global panel of 12 diverse isolates, with MLV included as a negative control (Fig. 3B). Half-maximal inhibitory dilution (ID_50_) values were calculated from percent neutralization upon fitting (Fig. S4). Consistent with our previous findings (37), the FR NP, BG5050 gp140.664.R1-FR, elicited a more potent autologous tier 2 NAb response than the soluble trimer, BG505 gp140.664.R1, with ID_50_ values of 222 to 783 for three of four rabbits, whereas only one of four rabbits in the trimer group yielded a detectable ID_50_ value (192) using a 100-fold starting dilution. Week 30 plasma from both groups neutralized clade A p398F1, whereas the trimer group had a more consistent pattern of neutralization, albeit very weak, against a clade A/E recombinant strain (pCNE8) using a 40-fold starting dilution. Week 30 rabbit plasma potently neutralized tier 1 clade B SF162, without nonspecific MLV reactivity. As another control, preimmunization samples (−day 10) were tested against the global panel in TZM-bl assays, which exhibited a clean background with no preexisting anti-HIV-1 activity (Fig. S4).

10.1128/mBio.00429-21.4 FIG S4 Rabbit plasma neutralization from two BG505 Env-immunized rabbit groups. Download FIG S4, PDF file, 1.0 MB. Copyright © 2021 Kumar et al. 2021 Kumar et al. https://creativecommons.org/licenses/by/4.0/ This content is distributed under the terms of the Creative Commons Attribution 4.0 International license.

Based on sample availability, we selected rabbit 35 (RB35) from the soluble trimer group and RB63 from the FR NP group for antibody isolation. Using the biotinylated Avi-tagged HR1-redesigned BG505 trimer probe (68, 69), we isolated Env-specific single B cells from PBMCs. A panel of rabbit MAbs was reconstituted from cloned HCs and KCs using a previously reported protocol (55), producing 34 and 55 HC-KC pairs for RB35 and RB63, respectively (Fig. S5A). Rapid functional screening based on antibody yield and BG505.T332N neutralization resulted in three hits, one from RB35 and two from RB63 (Fig. S5A). Sequence analysis revealed diverse germ line gene usage (Fig. S5B). RB35-1B11 is derived from IGHV1S45*01 and IGKV1S36*01, and RB63-1E7 and RB63-4B5 use the same HC germ line gene (IGHV1S40*01). Their KCs have an IGKV1S10*01 and IGKV1S15*01 origin, respectively. In the ELISA, the three rabbit NAbs were tested against the BG505 UFO.664 trimer (19) and four epitope probes, including a BG505 gp120-FR (41), an N332-I3-01 NP termed 1GUT_A_ES-I3-01 (37), a trimeric scaffold (PDB ID: 1TD0) presenting ZM109 V1V2 (termed ZM109 V1V2-5GS-1TD0), and an FP scaffold (termed FP-5GS-1TD0). All three rabbit NAbs showed high affinity for the trimer and gp120 probes (Fig. 3C, left) but no detectable binding to the N332, V1V2, and FP probes (Fig. S5C), suggesting that they recognize other epitopes in gp120 of BG505 Env. All three NAbs neutralized the autologous tier 2 clade A BG505.T332N but not the tier 1 clade B SF162 or negative control, MLV (Fig. 3C, right; Fig. S5D). Notably, the most potent rabbit NAb, RB63-4B5, yielded an IC_50_ of 0.022 μg/ml, which was ∼3-fold and 5-fold lower than the IC_50_ of mouse NAb M4H2K1 and the previously identified glycan hole NAbs (55), respectively. Last, all three rabbit NAbs showed negligible neutralization against the 12-virus global panel (Fig. S5E). Six non-NAbs, three from each rabbit, were confirmed to be nonreactive with BG505.T332N in the TZM-bl assays (Fig. S5F).

10.1128/mBio.00429-21.5 FIG S5 Functional evaluation of single-cell-sorted rabbit MAbs. Download FIG S5, PDF file, 0.5 MB. Copyright © 2021 Kumar et al. 2021 Kumar et al. https://creativecommons.org/licenses/by/4.0/ This content is distributed under the terms of the Creative Commons Attribution 4.0 International license.

To examine B-cell lineages associated with these three NAbs, we applied NGS to analyze Env-specific B cells from RB35 and RB63. Using the HR1-redesigned trimer probe, we sorted 363 and 370 Env-specific B cells from RB35 and RB63, respectively (Fig. S6A). Unbiased rabbit antibody HC and KC libraries were constructed for sequencing on the Ion S5 platform using a 5′-rapid amplification of cDNA reads (RACE) PCR protocol (86). NGS produced ∼1.1 and 1.9 million raw reads for RB35 and RB63, respectively, providing sufficient coverage for both HC and KC repertoires after processing using a rabbit antibodyomics pipeline (Fig. S6B). B-cell repertoire profiles revealed the focused HC germ line gene usage of IGHV1S40 (>22%), IGHV1S45 (>46%), and IGHV1S47 (>6%), accompanied by a broader and more diverse distribution of KC germ line genes (Fig. S6C). Notably, RB63, which was immunized with a BG505 gp140.664.R1-FR NP, showed a higher degree of SHM for KCs than RB35, which was immunized with a soluble BG505 trimer (Fig. S6C). Additionally, RB63 appeared to generate a large percentage (∼50%) of the B-cell lineage with a much longer (22-aa) HCDR3 loop (Fig. S6C). We then investigated the lineage prevalence of three potent rabbit NAbs and four non-NAbs (two per rabbit) within the NGS-derived repertoires (Fig. 3D and Fig. S6D). Using a CDR3 identity cutoff of 95% (90% for RB63-4B5), putative somatic variants were identified for the HC and KC of each antibody. All three NAbs exhibited reasonable lineage size, indicated by the distribution of CDR3-defined somatic variants on the 2D plots, whereas non-NAbs showed either no somatic variants or a highly expanded population, suggesting that they either were nonspecific Env binders or were induced by Env vaccination but failed to achieve any neutralizing activity during maturation.

10.1128/mBio.00429-21.6 FIG S6 HIV-1 Env-specific sorting and NGS of rabbit B cells for antibody isolation. Download FIG S6, PDF file, 0.7 MB. Copyright © 2021 Kumar et al. 2021 Kumar et al. https://creativecommons.org/licenses/by/4.0/ This content is distributed under the terms of the Creative Commons Attribution 4.0 International license.

Last, we examined whether these potent autologous NAbs target the previously identified glycan holes. We created a set of BG505.T332N Envs that had Q130N, D230N/K232T, S241N, P291T, and T465N mutations (55, 58). Neutralization by three rabbit NAbs was tested in the TZM-bl assay against these BG505.T332N mutants, except for D230N/K232T, which was not included because of the low yield of pseudoparticles (Fig. 3E). Among the four glycan hole mutations, Q130N did not affect HIV-1 neutralization by any of the three rabbit NAbs. In contrast, S241N and P291T completely abrogated neutralization by RB35-1B11 and RB63-1E7 but not for the more potent RB63-4B5, whereas T465N significantly reduced the potency of RB63-4B5 (IC_50_ > 5.0 μg/ml), confirming that these three NAbs targeted glycan holes at positions 241/289 and 465 that were reported in previous rabbit studies (55, 58). We then performed TZM-bl assays to investigate the prevalence of these glycan hole NAbs in the total polyclonal NAb response. Plasma neutralization against the four BG505.T332N mutants demonstrated that filling glycan holes could partially deplete neutralizing activity (Fig. 3F and Fig. S6E). Notably, the trimer group exhibited a more visible reduction when glycan holes at 241/289 were filled, suggesting that the trimer-induced autologous NAb response mainly targeted these two specific sites. Multivalent display on the FR 24-mer was able to expand this autologous NAb response to other sites on BG505 Env, consistent with recent findings that autologous rabbit NAbs could recognize various epitopes beyond glycan holes (57, 59). However, the FR-based immunogen failed to generate a detectable NAb response to the majority of tier 2 isolates in the global panel.

By combining single-cell NAb isolation with functional evaluation and repertoire NGS, we found that a gp41-stabilized BG505 trimer and its FR NP elicited potent autologous tier 2 NAbs, most of which targeted previously identified glycan holes. Remaining unclear, however, is whether the E2p and I3-01 60-mers (37, 41), which are 23 to 25 nm in diameter, can broaden the Env-induced NAb response more effectively than the FR 24-mer, which is only ∼12 nm. The EM-based epitope mapping method (61) may be used in future studies to identify other sites on HIV-1 Env that are recognized by trimer and NP-induced polyclonal antibody responses in rabbit immunization.

### Characterization of tier 2 clade A and clade C T/F Env immunogens *in vitro* and *in vivo*.

BG505 trimers, regardless of the design and display platforms, mostly induced glycan hole NAbs in rabbits (55). Therefore, other HIV-1 Envs that are able to elicit a broader NAb response during immunization need to be identified. Clade C viruses are important because they are responsible for approximately half of global infections (87). NFL trimers have been designed and structurally characterized for a tier 2 clade C T/F strain, 16055 (31). In rabbits, the 16055 NFL trimers that were arrayed on liposome NPs elicited a moderate autologous NAb response with V2 specificity (42) and generated a bNAb response to the CD4bs when immunized with a heterologous regimen (30). We previously reported gp41-stabilized trimers for clade A Q842.d12 and clade C Du172.17, both of which are tier 2 T/F strains (19, 37). In the present study, we assessed the vaccine potential of these two T/F Envs compared with previously characterized BG505 and 16055 Envs.

**FIG 4:**
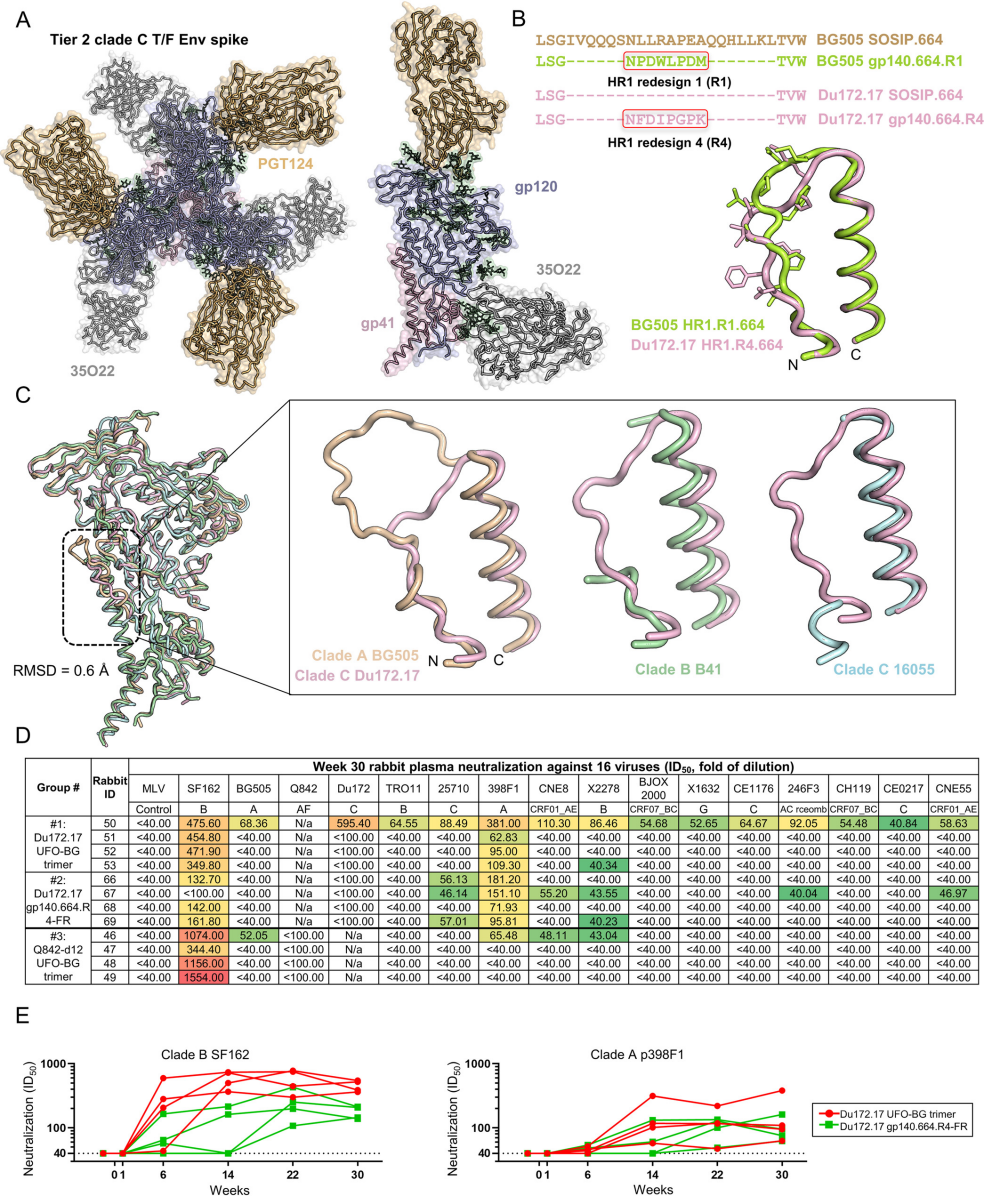
Structure of clade C Du172.17 Env and evaluation of three Env immunogens in rabbits. (A) Crystal structure of closed prefusion structure of Du172.17 gp140.664.R4 Env trimer, which is uncleaved and contains a computationally redesigned HR1 (HR1-#4 [19]). Top view of the Du172.17 Env-Fab complex along the trimer axis with gp120 in blue and gp41 in pink. Side view of the Du172.17 Env protomer bound to Fabs PGT124 (orange) and 35O22 (dark gray) from the 3.4-Å-resolution crystal structure. The cartoon representation is overlaid with the transparent molecular surface. (B) Sequence and structural alignment of the N terminus of the HR1 region (HR1_N_) in two trimer designs: BG505 gp140.664.R1 and Du172.17 gp140.664.R4. The redesigned 8-residue HR1 region is highlighted to facilitate comparison. (C) Superimposition of cleaved Du172.17 gp140.664.R4 (pink), cleaved BG505 SOSIP.664 (orange; PDB ID: 5CEZ), cleaved B41 SOSIP.664 (green; PDB ID: 6MDT), and uncleaved 16055 NFL.664 (cyan; PDB ID: 6P65) protomers. The inset on the right shows a closeup view of Du172.17 HR1_N_ superimposed onto HR1_N_ from each of the three Envs. (D) Neutralization (measured by ID_50_ values) of week 30 plasma from three rabbit groups against two respective autologous viruses (Du172.17 and Q842-d12), tier 2 clade A BG505.T332N, tier 1 clade B SF162, and a 12-virus global panel, with MLV included as a negative control. Color coding indicates neutralization potency (red, potent; green, neutralizing but not potent; no color, nonneutralizing). (E) Longitudinal analysis of plasma from two rabbit groups immunized with Du172.17 trimer and FR NP at six time points against tier 1 clade B SF162 (left) and tier 2 clade A p398F1 (right). Rabbits from the trimer and FR groups are shown as red and green lines, respectively.

We first sought to determine atomic structures for these two Envs. Well-ordered crystals were obtained only for one Du172.17 Env construct, which is cleaved and contains a redesigned HR1_N_ bend (HR1-#4) (19). This construct, termed Du172.17 gp140.664.R4, was expressed in HEK293S cells and purified on a 2G12 affinity column (14) before adding Fabs PGT124 and 35O22 to aid crystallization. The Fab-bound Du172.17 Env trimer complex crystallized at 20°C, and its structure was determined at 3.40-Å resolution in a hexagonal (P6_3_) crystal lattice (Fig. 4A). The HR1_N_ region in this construct was designed specifically to stabilize the prefusion Du172.17 Env (19) and thus is different from the HR1_N_ region that was designed for BG505 Env (Fig. 4B). Little difference was observed in the overall Env structure (Cα root mean square deviation [RMSD] = 0.6 Å) between BG505 gp140.664.R1 and Du172.17 gp140.664.R4 except in the 8-aa HR1_N_ segment (Fig. 4C). To further evaluate the difference in overall Env conformation and the redesigned HR1_N_, we superimposed the Du172.172 protomer onto crystal structures that were previously determined for clade A, B, and C Envs (Fig. 4C). As expected, Du172.17 gp140.664.R4 adopted a protomer structure similar to BG505 SOSIP.664 (79), B41 SOSIP.664 (88), and 16055 NFL.664 (31) with a Cα RMSD of 0.6 Å. Nevertheless, a large conformational change in HR1_N_ was observed between Du172.17 and BG505. Comparisons with the other Envs with their native-like full-length HR1 were not possible because of disorder in the HR1_N_ helical region in their crystal structures. We also superimposed the Du172.17 gp140.664.R4 protomer onto crystal and cryo-EM structures of several clade C Envs (PDB ID: 5UM8 [31]; PDB ID: 6P65 [30]; PDB ID: 6MYY [89]; PDB ID: 6UM6 [90]). Although the sequence identity among these clade C Envs is 76 to 78%, they share high structural similarity with Cα RMSDs of 0.7 to 1.3 Å (Fig. S7). Altogether, the low Cα RMSD values observed for SOSIP, NFL, and HR1-redesigned trimers suggest that the HR1_N_ modification has no adverse impact on the overall architecture and compactness of this clade C Env.

10.1128/mBio.00429-21.7 FIG S7 Structural comparison of HIV-1 Envs across clade C isolates. Download FIG S7, PDF file, 0.8 MB. Copyright © 2021 Kumar et al. 2021 Kumar et al. https://creativecommons.org/licenses/by/4.0/ This content is distributed under the terms of the Creative Commons Attribution 4.0 International license.

After confirming that the HR1-redesgined Du172.17 Env adopts a native-like, prefusion trimer conformation, we next compared glycosylation patterns of SOSIP, HR1-redesigned, and UFO trimers for this clade C Env using the same strategy previously reported for BG505 Env (37). Briefly, HEK293F-expressed proteins were harvested from media and purified on a PGT145 column (15) followed by size exclusion chromatography (SEC) on a Superdex 200 column. Liquid chromatography-mass spectrometry (LC-MS) was employed to determine site-specific glycosylation (Fig. S8), which was enabled by digesting the Env protein into peptides and glycopeptides using three separate proteases: trypsin, chymotrypsin, and elastase. The relative proportions of different glycans were determined and grouped to facilitate comparisons between samples (Fig. S8). The key features that define native-like glycosylated trimers include high mannose content and occupancy at each site. The majority of N-linked glycosylation sites on all three trimers contain large amounts of oligomannose-type glycans. Conserved glycan sites across HIV-1 strains that presented oligomannose-type glycans include the N332 supersite and apex glycan N160. All three design formats have ∼100% occupancy at these sites with oligomannose-type glycans, consistent with a well-folded native-like trimer. The complex-type glycans that are observed across the samples are fucosylated bi- and triantennary glycans that are common in HEK293F and CHO cells. Occupancy at every site in all three trimers was greater than 95%, except for N611. However, glycan holes may still be present at sites that could not be resolved in this analysis (Fig. S8). Some regions in the gp41 ectodomain (gp41_ECTO_) showed significant deviation in glycosylation patterns. No oligomannose-type glycans were observed at N611 on the SOSIP trimer, but high mannose content was observed on the HR1-redesigned and UFO trimers (67% and 24%, respectively). Likewise, the SOSIP and HR1-redesigned trimers contained 39% and 51% oligomannose-type glycans at N625, respectively, whereas the UFO trimer had no oligomannose-type glycans at this site. Our data suggest that steric restrictions imposed upon glycan sites by surrounding protein regions differ slightly (e.g., with or without a linker at the cleavage site), and most epitopes in gp120 and at the gp120-gp41 interface are unaffected by the design platform.

10.1128/mBio.00429-21.8 FIG S8 Site-specific N-linked glycan analysis of Du172.17 SOSIP.664, HR1-redesigned (gp140.664.R4), and UFO.664 trimers produced in HEK293F cells. Download FIG S8, PDF file, 0.09 MB. Copyright © 2021 Kumar et al. 2021 Kumar et al. https://creativecommons.org/licenses/by/4.0/ This content is distributed under the terms of the Creative Commons Attribution 4.0 International license.

Last, we immunized three groups of rabbits to assess the immunogenicity of a Du172.17 trimer, a Du172.17 gp140-FR NP, and a Q842.d12 trimer. For Du172.17, we tested a UFO-BG trimer, which contained Du172.17 gp120 and BG505 gp41_ECTO_ of the UFO design (37) and an FR NP presenting the structurally defined gp140.664.R4 trimer (19). For Q842.d12, a UFO-BG trimer was selected for consistency with Du172.17. The same regimen was used for rabbit immunization (Fig. 3A) to facilitate comparisons with the previous study of BG505 (37). We first assessed rabbit plasma at the last time point (week 30) against an expanded panel of viruses, including the respective autologous virus (either Du172.17 or Q842-d12), BG505.T332N, tier 1 SF162, and the 12-virus global panel, with MLV used as a control (Fig. 4D and Fig. S9A to F). Overall, distinct neutralization patterns were observed compared with previous rabbit studies. Using a 100-fold starting dilution, autologous NAb responses were not observed for any group except for plasma from RB50 in the Du172.17 trimer group, which also neutralized all tested HIV-1 isolates and exhibited a detectable nonspecific response to MLV (Fig. S9A). Notably, the lack of autologous neutralization was also found for the 16055 NFL trimer (42) and BG505 gp140.664.R1 trimer (Fig. 3B) (37). In contrast, a robust autologous NAb response was observed when these trimers were conjugated to liposome NPs (42) or displayed on FR NPs (Fig. 3B) (37). Unexpectedly, multivalent display did not improve the autologous NAb response for Du172.17 Env (Fig. 4D). Nonetheless, the Du172.17 trimer and FR NP elicited consistently, albeit slightly, stronger NAb responses to clade A p398F1 compared with their BG505 counterparts, using a 40-fold starting dilution (Fig. 3B and Fig. 4D). Borderline neutralization was detected against other isolates, such as clade C p25710 and clade B pX2278, in some rabbits. When tested against tier 1 clade B SF162, the Q842-d12 trimer showed the highest NAb titers (ID_50_ = 344 to 1,554), whereas the Du172.17 gp140.664.R4-FR NP yielded ID_50_ values of 162 or lower. Preimmunization (−day 10) samples exhibited negligible reactivity on the 12-virus panel, indicating a clean background (Fig. S9A to F). Last, we characterized longitudinal NAb development in the Du172.17 trimer and FR NP groups (Fig. 4E and Fig. S9G). The soluble trimer elicited a more rapid tier 1 NAb response to clade B SF162 than the gp140-FR NP, and for most time points, this response also appeared to be more potent than the NP-induced tier 1 NAb response (Fig. 4E, left). In contrast, the tier 2 NAb response to clade A p398F1 exhibited a distinct pattern, with measurable plasma neutralization observed only for week 14 and onward (Fig. 4E, right). The difference in such a tier 2 response was not significant between the trimer and FR groups, likely because of the small group size and outlier (RB50, with preexisting Env-reactive B cells) in the trimer group.

10.1128/mBio.00429-21.9 FIG S9 Rabbit plasma neutralization from three non-BG505 Env-immunized rabbit groups. Download FIG S9, PDF file, 1.9 MB. Copyright © 2021 Kumar et al. 2021 Kumar et al. https://creativecommons.org/licenses/by/4.0/ This content is distributed under the terms of the Creative Commons Attribution 4.0 International license.

The crystal structure of a novel tier 2 clade C T/F Env confirmed the effectiveness of HR1 redesign in Env stabilization and provided a structurally defined, native-like trimer for NP display and *in vivo* assessment. The rabbit study of three Env immunogens that were derived from two T/F Envs of clades A and C showed limited improvement in the NAb response. The clade A Q842-d12 UFO-BG trimer, despite having excellent *in vitro* properties (37), generated primarily a tier 1 NAb response. The FR NP display of a native-like BG505 trimer improved the autologous NAb response (37), but this strategy showed little success for Du172.17. Altogether, the elicitation of a robust NAb response to diverse tier 2 isolates remains a challenge for HIV-1 vaccine design.

## DISCUSSION

HIV-1 vaccine development has entered a new era since the demonstration of tier 2 NAb responses in rabbits and NHPs elicited by the vaccination of prototypic native-like SOSIP trimers (51, 91). The success of the BG505 SOSIP trimer as a structural template to study bNAb-Env interactions and as an Env backbone to experiment with various rational design concepts has led to a plethora of studies that position native-like Env trimers at the center of HIV-1 vaccine research (12, 20). However, the goal of eliciting a bNAb response upon vaccination remains elusive, and a number of fundamental questions related to the inherent features of HIV-1 Env need to be addressed in the next phase of HIV-1 vaccine development (92). In recent studies, we have demonstrated a vaccine strategy by combining antigen design/optimization and NP display and have applied this strategy to HIV-1 (19, 41), hepatitis C virus (HCV) (93), Ebola virus (EBOV) (94), and SARS-CoV-2 (95). One of our first-generation HIV-1 immunogens, a 60-meric I3-01 NP presenting 20 gp41-stabilized BG505 gp140 trimers, elicited a tier 2 NAb response in wild-type mice (37), which was not previously found to be possible for the BG505 SOSIP.664 trimer (45). However, critical issues about trimer design, NP display, and animal models remain open questions in HIV-1 vaccine development. Insights into these issues for HIV-1, which is arguably the most challenging virus vaccine target to date, will continue to inform and facilitate vaccine development efforts for other viruses.

Here, we addressed some of these issues for HIV-1 by analyzing animal samples from our previous study (37) and by testing new Env immunogens in rabbits. First, we provided evidence at the monoclonal level that potent tier 2 NAbs can be elicited in wild-type mice using a multivalent Env immunogen. This finding is critical because it highlights the advantage of our vaccine strategy and touches on an important question in the HIV-1 field—whether native-like Env can elicit tier 2 NAbs in mice, which has not been addressed with clarity. In this study, the nsEM model and crystal structure of NAb M4H2K1 in complex with BG505 Env identified a target for potent mouse NAbs, which is also an epitope for the autologous NAb response in early human infection (5). In mouse immunization studies, the nonspecific antiviral component in serum poses a challenge for HIV-1 pseudovirus assays and has been the source of inconsistencies (37, 44, 45, 96). To overcome this problem, IgG must be purified from serum samples to unambiguously demonstrate the elicitation of tier 2 NAbs in mice, as shown in our previous study (37). Altogether, our results indicate that wild-type mice are a suitable animal model for screening HIV-1 Env immunogens and probing their immunologic mechanisms, as demonstrated in recent studies of multivalently displayed Envs in mice (96–98). The difficulty of eliciting NAbs in wild-type mice appears to be unique to HIV-1 and has not been observed for other viruses in our vaccine studies (93–95). Second, we provided evidence at the monoclonal level that BG505 Env containing a redesigned HR1_N_ segment (the core of the UFO trimer design) (19), both as a soluble trimer and displayed on a 24-meric FR NP, can elicit a potent tier 2 NAb response that is primarily directed to known glycan holes (55, 59). The display of BG505 trimers on this small protein NP did not broaden the autologous NAb response in rabbits (Fig. 3B) (37). These results, together with recent findings from a rabbit study of mixed BG505 and B41 SOSIP trimers (85), highlight a key limitation of the rabbit model for evaluating HIV-1 Env vaccines. Nonetheless, FR display did appear to drive the NAb response away from glycan holes, as shown in the TZM-bl assays against glycan hole mutants (Fig. 3F), suggesting a positive effect of NP display on bNAb elicitation. Therefore, the 60-meric E2p and I3-01 NPs may warrant further investigation because their large size will likely enhance this effect and result in a broader NAb response. Indeed, we observed a more effective cross-neutralizing response for E2p and I3-01 NPs in recent HCV, EBOV, and SARS-CoV-2 vaccine studies (93–95, 99). Third, we demonstrated that our rational design strategy can readily generate stable, native-like trimers for diverse Envs, as shown for clade A Q842-d12 and clade C Du172.17, although eliciting a bNAb response remains a significant challenge. Nonetheless, the crystal structure of Du172.17 Env may serve as a useful template for designing clade C-specific vaccines. Substantial differences in NAb responses elicited by Env trimers of the same clade (e.g., BG505 versus Q842-d12) also suggest that the ability to elicit a NAb response and the breadth and potency of this response may be encoded in specific features of the Env sequence, thus highlighting the necessity to screen diverse Envs to facilitate future HIV-1 vaccine development. Notably, this preferential NAb response is not unique to HIV-1. In our recent study, the SARS-CoV-2 spike appeared to be far more effective than the SARS-CoV-1 spike in eliciting a potent NAb response to both SARS-CoVs (95).

Several directions may be explored in our future HIV-1 vaccine research. First, more advanced NP platforms may be used to display the stabilized Env trimers. Recently, we developed multilayered protein NPs based on E2p and I3-01 60-mers, which were used to present stabilized EBOV and SARS-CoV-2 glycoproteins (94, 95). In terms of stability and manufacturability, these two reengineered NPs may be more advantageous than other NP platforms (100, 101) because of their superior stability and high expression in good manufacturing practice (GMP)-compatible CHO cells. Second, a more in-depth immunological analysis of the vaccine mechanism may be needed for HIV-1 trimer-presenting NPs, as demonstrated for other HIV-1 vaccine candidates (96–98, 102). Recently, we investigated adjuvant effects, trafficking, retention, presentation, and germinal center reactions for a COVID-19 NP vaccine that elicits a bNAb response to SARS-CoV-2 variants (99). As we began to switch to our more advanced UFO trimer design (37) for future HIV-1 vaccine development, critical questions related to HIV-1 Env (92) and vaccine mechanisms can be pursued in the context of UFO trimers and UFO trimer-presenting NPs.

## MATERIALS AND METHODS

### Expression and purification of HIV-1 Env probes, trimers, and gp140 nanoparticles.

The Avi-tagged BG505 gp140.664.R1 trimer probe was transiently expressed in HEK293F cells (Thermo Fisher) (68). Env protein was purified from the supernatant by a Galanthus nivalis lectin (GNL) column (Vector Labs) and eluted with PBS containing 500 mM NaCl and 1 M methyl-α-d-mannopyranoside. Biotinylation was performed using the BirA biotin-protein ligase standard reaction kit (BirA-500) according to the manufacturer’s instructions (Avidity). This BG505 trimer probe was further purified by SEC on a HiLoad 16/600 Superdex 200 PG column (GE Healthcare). A FR NP presenting BG505 V1V2 and a trimeric scaffold (1TD0) presenting ZM109 V1V2 were transiently expressed in *N*-acetylglucosaminyltransferase I-negative (GnTI^−/−^) HEK293S cells (Thermo Fisher) (41). Both FR and I3-01 NPs presenting an N332-scaffold, 1GUT_A_ES, were transiently expressed in HEK293F cells treated with kifunensine (Tocris Bioscience) (70). Both V1V2 and N332 epitope probes were extracted from the supernatant using a GNL column. Fusion peptide (FP) probes were created by fusing the FP motif, AVGIGAVFL, to an FR or 1TD0 subunit with a 5GS (G_4_S) linker. The FP-5GS-FR and BG505 gp120-FR probes were transiently expressed in ExpiCHO cells (Thermo Fisher) using a protocol similar to that for BG505 gp140 NPs (37). The trimeric FP-5GS-1TD0 probe was also transiently expressed in ExpiCHO cells. Immunoaffinity columns based on bNAbs VRC34 (103) and PGT145 (104) were used to extract the two FP probes (FP-5GS-FR NP and FP-5GS-1TD0 trimer) and the BG505 gp120-FR NP probe from the supernatant, respectively. After purification using a GNL or antibody column, the NP and 1TD0-derived epitope probes were further purified by SEC on a Superose 6 10/300 GL column and a Superdex 75 10/300 GL column (GE Healthcare), respectively. For rabbit immunization, the Du172.17 UFO-BG trimer, the FR NP presenting a structurally defined Du172.17 gp140.664.R4 trimer, which is cleaved and contains a redesigned HR1 (HR1-#4) (19), and the Q842-d12 UFO-BG trimer were transiently expressed in ExpiCHO cells (37). For the two UFO-BG trimers, Env protein was extracted from the supernatant by a GNL column and then purified by SEC on a HiLoad 16/600 Superdex 200 PG column. The Du172.17 gp140.664.R4-FR NP was purified using a 2G12 affinity column (14) followed by SEC on a Superose 6 10/300 GL column. Protein concentrations were determined using UV absorbance at 280 nm (UV_280_) with theoretical extinction coefficients.

### Env-specific sorting of mouse and rabbit B cells.

Mouse spleen cells harvested 15 days after the last injection were prepared for sorting. Cells were first stained for the exclusion of dead cells with Fixable Aqua dead cell stain (Thermo Fisher). Receptors FcγIII (CD16) and FcγII (CD32) were blocked by 20 μl of 2.4G2 MAb (BD Pharmingen). Cells were then incubated with 10 μg of biotinylated Avi-tagged BG505 gp140.664.R1 trimer probe for 5 min at 4°C, followed by the addition of 2.5 μl of anti-mouse IgG fluorescently labeled with fluorescein isothiocyanate (FITC) (Jackson ImmunoResearch) and incubated for 15 min at 4°C. Finally, 5 μl of premium-grade allophycocyanin (APC)-labeled streptavidin (Thermo Fisher) was added to the cells and incubated for 15 min at 4°C. In each step, cells were washed with 500 μl of fluorescence-activated cell sorting (FACS) buffer (Dulbecco’s phosphate-buffered saline [DPBS] with 2% fetal bovine serum [FBS]). FITC^+^ APC^+^ Env-specific B cells were sorted using MoFloAstrios EQ (Beckman Coulter). Rabbit PBMCs obtained 30 days after the last injection were prepared for sorting. After staining for the exclusion of dead cells with Fixable Aqua dead cell stain (Thermo Fisher), cells were incubated with 10 μg of biotinylated Avi-tagged BG505 gp140.664.R1 trimer probe for 5 min at 4°C, followed by the addition of 2 μl of anti-rabbit IgG conjugated with DyLight 405 (Jackson ImmunoResearch), 2 μl of anti-rabbit T lymphocytes fluorescently labeled with FITC (Bio-Rad), and 2 μl of anti-rabbit IgM fluorescently labeled with FITC (Bio-Rad), and then incubated for 15 min at 4°C. Finally, 5 μl of APC-labeled streptavidin (Thermo Fisher) was added to the cells and incubated for 15 min at 4°C. In each step, cells were washed with 500 μl FACS buffer (DPBS with 2% FBS). FITC^−^ DyLight 405^+^ APC^+^ Env-specific B cells were sorted using MoFloAstrios EQ (Beckman Coulter). For bulk sorting, positive cells were sorted into an Eppendorf microtube with 20 μl of lysis buffer. For single B-cell sorting, individual positive cells were sorted into the inner wells of a 96-well plate with 20 μl of a pre-reverse-transcription (RT) lysis mix containing 0.1 μl of NP-40 (Sigma-Aldrich), 0.5 μl of RNase inhibitor (Thermo Fisher), 5 μl of 5× first-strand buffer, 1.25 μl of dithiothreitol (DTT) from the SuperScript IV kit (Invitrogen), and 13.15 μl of H_2_O per well.

### Antibody cloning from Env-specific single B cells and antibody production.

The antibody cloning of Env-sorted single B cells was conducted as follows. A mix containing 3 μl of random hexamers (GeneLink), 2 μl of deoxynucleoside triphosphates (dNTPs), and 1 μl of SuperScript IV enzyme (Thermo Fisher) was added to each well of a single-cell-sorted 96-well plate that underwent thermocycling according to the program outlined in the SuperScript IV protocol, resulting in 25 μl of cDNA for each single cell. cDNA (5 μl) was then added to a PCR mix containing 12.5 μl of 2× multiplex PCR mix (Qiagen), 9 μl of H_2_O, 0.5 μl of forward primer mix, and 0.5 μl of reverse primer mix (mouse [105] and rabbit [55]) for HCs and KCs within each well. A second PCR was then performed using 5 μl of the first PCR as the template and respective primers (mouse [105] and rabbit [55]) utilizing the same recipe as the first PCR. The PCR products were run on a 1% agarose gel, and those with correct HC and KC bands were then used for Gibson ligation (New England Biolabs), cloning into IgG expression vectors, and transformation into competent cells. Mouse and rabbit MAbs were expressed by the transient transfection of ExpiCHO cells (Thermo Fisher) with equal amounst of paired HC and KC plasmids and purified from the culture supernatant after 12 to 14 days using protein A bead columns (Thermo Fisher).

### NGS and bioinformatics analysis of mouse and rabbit B cells.

We combined the 5′-rapid amplification of cDNA ends (RACE) protocol with previously reported HC and KC primers for mouse (105) and rabbit (86) to facilitate the NGS analysis of Env-specific mouse splenic B cells and rabbit B cells, respectively. Briefly, 5′-RACE cDNA was obtained from bulk-sorted B cells of each animal with the SMART-Seq v4 ultralow-input RNA kit for sequencing (TaKaRa). The Ig PCRs were set up with Platinum *Taq* high-fidelity DNA polymerase (Thermo Fisher) in a total volume of 50 μl, with 5 μl of cDNA as the template, 1 μl of 5′-RACE primer, and 1 μl of 10 μM reverse primer. The 5′-RACE primer contained a PGM/S5 P1 adaptor, and the reverse primer contained a PGM/S5 A adaptor. For mouse samples, we adapted the mouse 3′-C_γ_1-3/3′-C_μ_ inner primers and 3′-mC_κ_ outer primer (105) as reverse primers for 5′-RACE PCR processing of HCs and KCs, respectively. For rabbit samples, we adapted rabbit RIGHC1/RIGHC2 primers and RIGkC primers (86) as reverse primers for the 5′-RACE PCR processing of HCs and KCs, respectively. A total of 25 cycles of PCR were performed, and the expected PCR products (500 to 600 bp) were gel purified (Qiagen). NGS was performed on the Ion S5 GeneStudio platform. Briefly, HC and KC libraries from the same animal were quantitated using a Qubit 2.0 fluorometer with the Qubit double-stranded DNA (dsDNA) high-sensitivity (HS) assay kit and then mixed at a ratio of 2:1 or 3:1 before being pooled with antibody libraries from the other animals at an equal ratio. Template preparation and Ion 530 chip loading were performed on Ion Chef using the Ion 520/530 Ext kit, followed by sequencing on the Ion S5 system with default settings. The mouse antibodyomics pipeline (70) was used to process the mouse NGS data. The rabbit antibodyomics pipeline was created by incorporating rabbit germ line genes from IMGT (http://www.imgt.org/) into the reference libraries. Quantitative repertoire profiles were generated for germ line gene usage, the degree of SHM, and H/KCDR3 loop length. Two-dimensional (2D) divergence/identity plots were generated to visualize selected mouse and rabbit NAb/MAb chains in the context of Env-specific B-cell repertoires. A previously described sequence clustering algorithm (68) was used to derive consensus HCs and KCs for prevalent antibody lineages from the NGS data of bulk-sorted mouse splenic B cells. NGS-derived MAbs were transiently expressed in ExpiCHO cells (Thermo Fisher) with equal amounts of HC and KC plasmids and purified from culture supernatants after 12 to 14 days using protein A bead columns (Thermo Fisher).

### Enzyme-linked immunosorbent assay.

Each well of a Costar 96-well assay plate (Corning) was first coated with 50 μl of PBS containing 0.2 μg of appropriate antigens. The plates were incubated overnight at 4°C and then washed five times with wash buffer containing PBS and 0.05% (vol/vol) Tween 20. Each well was then coated with 150 μl of blocking buffer consisting of PBS, 40 mg/ml blotting-grade blocker (Bio-Rad), and 5% (vol/vol) FBS. The plates were incubated with blocking buffer for 1 h at room temperature and then washed five times with wash buffer. For antigen binding, antibodies were diluted in blocking buffer to a maximum concentration of 10 μg ml^−1^, followed by a 10-fold serial dilution. For each antibody dilution, 50 μl was added to the appropriate wells. Next, a 1:5,000 dilution of goat anti-human IgG antibody (Jackson ImmunoResearch Laboratories) was made in the wash buffer (PBS containing 0.05% Tween 20), with 50 μl of the diluted secondary antibody added to each well. The plates were incubated with the secondary antibody for 1 h at room temperature and then washed five times with PBS containing 0.05% Tween 20. Finally, the wells were developed with 50 μl of 3,3′,5,5′-tetramethylbenzidine (TMB) (Thermo Fisher) for 3 to 5 min before stopping the reaction with 50 μl of 2 N sulfuric acid. The resulting plate readouts were measured at a wavelength of 450 nm. The EC_50_ values were calculated in GraphPad Prism 8.4.3.

### Pseudovirus production and neutralization assays.

Pseudoviruses were generated by the transfection of HEK293T cells with an HIV-1 Env-expressing plasmid and an Env-deficient genomic backbone plasmid (pSG3ΔEnv), as previously described (106). HIV-1 Env-expressing vectors for BG505 (catalog no. 11518), SF162 (catalog no. 10463), and the global panel (72) (catalog no. 12670) were obtained through the NIH AIDS Reagent Program (https://www.aidsreagent.org/). A T332N mutation was introduced into BG505 Env to produce the BG505.T332N clone. Other BG505.T332N mutants were created by introducing mutations as previously described (55, 59, 82). Pseudoviruses were harvested 72 h posttransfection for use in the neutralization assays. The neutralizing activity of heat-inactivated rabbit plasma was assessed using a single round of replication pseudovirus assay and TZM-bl target cells, as described previously (106). Briefly, pseudovirus was incubated with serial dilutions of antibodies or rabbit plasma in a 96-well flat-bottom plate for 1 h at 37°C before TZM-bl cells were seeded in the plate. For antibody neutralization, a starting concentration of 5 μg/μl was used and subjected to a 3-fold serial dilution in the TZM-bl assays. Rabbit plasma was diluted 100-fold and 40-fold against autologous and heterologous pseudoviruses, respectively, and then subjected to a 3-fold serial dilution in the TZM-bl assays. As a negative control, pseudoparticles displaying the envelope glycoproteins of MLV were tested in the TZM-bl assays following the same protocol. Luciferase reporter gene expression was quantified 48 to 72 h after infection upon lysis and the addition of Bright-Glo luciferase substrate (Promega). Data were retrieved from a BioTek microplate reader with Gen 5 software. Background luminescence from a series of uninfected wells was subtracted from each experimental well, and neutralization curves were generated using GraphPad Prism 8.4.3, in which values from experimental wells were compared against a well containing virus only. To determine IC_50_ and ID_50_ values, dose-response curves were fit by nonlinear regression in GraphPad Prism 8.4.3.

### Expression and purification of BG505 gp120 core, Du172.17 gp140, and Fabs and complex formation for structural analysis.

The antigen-binding fragments (Fabs) of M4H2K1, 17b, PGT124, and 35O22 were expressed in FreeStyle HEK293F cells (Invitrogen) and purified by CaptureSelect CH1-XL affinity (Thermo Fisher) chromatography followed by SEC on a Superdex 75 16/600 column (GE Healthcare). The BG505 gp120 core protein was transiently expressed in FreeStyle HEK293S cells and extracted from the supernatant using a GNL affinity column, followed by SEC on a Superdex 200 16/600 column (GE Healthcare). A complex was formed by combining gp120-M4H2K1-17b in a 1:2:2 molar ratio, followed by deglycosylation using endo H digestion (New England Biolabs) at 37°C for 1 h before SEC purification. The gp120:M4H2K1:17b complex was then analyzed by sodium dodecyl sulfate–polyacrylamide gel electrophoresis (SDS-PAGE). The clade C Du172.17 gp140.664.R4 Env trimer was expressed in FreeStyle HEK293S cells. Env protein was harvested from the medium and purified with a 2G12 column (14) followed by SEC on a Superdex 200 column (GE Healthcare). The Du172.17 trimer complex was formed by mixing PGT124 and 35O22 Fabs in a molar ratio of 1:3.5:3.5 (Du172-PGT124-35O22) at room temperature for 30 min. The trimer complex was partially deglycosylated using endo H digestion (New England Biolabs) (23) at 37°C for 1 h and then purified on a Superdex 200 column. The complex was SEC purified in 50 mM Tris-HCl and 150 mM NaCl (pH 7.4) and concentrated to ∼10 mg/ml prior to crystallization trials.

### Crystallization and data collection.

The SEC-purified Fab M4H2K1 and complex were each concentrated to 12 mg/ml before being screened at both 4°C and 20°C using our high-throughput CrystalMation robotic system (Rigaku) at The Scripps Research Institute (TSRI) (107). Crystals of unbound Fab M4H2K1 were grown in 0.1 M N-cyclohexyl-2-aminoethanesulfonic acid (CHES, pH 9.5) and 36% polyethylene glycol (PEG) 600 at 4°C. Crystals of the M4H2K1/gp120 core complex were grown in 0.1 M Tris (pH 7), 1.825 M ammonium sulfate, 0.29 M lithium sulfate, and 15% ethylene glycol at 20°C. Crystals were harvested and followed by immediate flash cooling in liquid nitrogen. The Du172.17 trimer complex was set up at both 4°C and 20°C using our Rigaku CrystalMation robotic system. High-quality crystals of Fabs PGT124 and 35O22 bound to the HR1-redesigned Du172.17 trimer were obtained in 0.1 M Tris (pH 8.4) and 25% (vol/vol) PEG 400 at 20°C. Data were collected at the Advanced Photon Source (APS) on beamlines 23-IDD and 23-IDB.

### Structure determination and refinement.

The unbound Fab M4H2K1 and Fab M4H2K1/BG505 gp120 core/Fab 17b crystals diffracted to 1.50-Å and 4.30-Å resolution, respectively. The data were indexed, integrated, and scaled using HKL2000 (108) in P3_1_21 for unbound M4H2K1-Fab and in P2_1_2_1_2 for the complex. The unbound Fab structure was solved by molecular replacement (MR) using Phaser (109) with Fab structures (PDB 5GS1 [110] for the variable region and PDB 5BZW [111] for the constant region) as MR search models. The BG505 gp120 core in complex with Fabs M4H2K1 and 17b was determined by MR using PDB 6ONF (112) for the gp120 core, the unbound Fab M4H2K1 structure for the bound Fab M4H2K1, and PDB 1GC1 (78) for Fab 17b as the search models. The unbound M4H2K1 Fab crystal structure was refined to *R*_cryst_/*R*_free_ of 18.3%/21.6% with 99.8% completeness and unit cell parameters *a *=* b *= 68.3 Å, *c *= 184.7 Å (see Table S1 in the supplemental material). The Fab M4H2K1-bound gp120 core complex structure was refined to *R*_cryst_/*R*_free_ of 30.1%/33.3% with 86.8% completeness and unit cell parameters *a* = 204.0 Å, *b* = 60.6 Å, *c* = 166.7 Å (Table S1). The Du172.17 trimer in complex with Fabs PGT124 and 35O22 crystal diffracted to 3.40 Å resolution and the diffraction data were processed (indexed, integrated and scaled) with HKL2000 in the P6_3_ space group. The Du172.17 trimer in complex with Fabs PGT124 and 35O22 was determined using PDB 5CEZ (79) for Env Du172.17 gp140, PDB 4TOY (113) for 35O22, and PDB 4R26 (114) for the Fab PGT124 structure as the MR search models. The crystal structure of the Du172.17 trimer complex was refined to *R*_cryst_/*R*_free_ of 24.2%/29.1% and overall completeness of 97.6% and unit cell parameters *a *=* b* = 127.0 Å, *c* = 316.5 Å (Table S1). Model building and refinement were carried out with Coot and Phenix, respectively (115–117). Structure quality was determined by MolProbity (118). The Kabat numbering scheme (119) was used for Fabs M4H2K1 and 17b. The BG505 gp120 core and Du172.17 trimer were numbered according to the HXB2 system (120). Structure validation was performed using the PDB Validation Server (https://validate.wwpdb.org), PDB-care (121), and Privateer (122). Data collection and refinement statistics are outlined in Table S1.

10.1128/mBio.00429-21.10 TABLE S1 X-ray crystallographic data collection and refinement statistics. Download Table S1, DOCX file, 0.04 MB. Copyright © 2021 Kumar et al. 2021 Kumar et al. https://creativecommons.org/licenses/by/4.0/ This content is distributed under the terms of the Creative Commons Attribution 4.0 International license.

### Negative-stain electron microscopy.

Complexes of M4H2K1 Fab and BG505 UFO.664 trimer were purified by SEC to remove unbound Fab and diluted to 0.01 mg/ml in Tris-buffered saline prior to adsorption onto carbon-coated and plasma-cleaned copper mesh grids (Cu400; Electron Microscopy Sciences). Grids were stained with 2% (wt/vol) uranyl formate for ∼60 s and imaged on an FEI Tecnai Spirit microscope operating at 120 keV, equipped with a TVIPS TemCam F416 4k × 4k complementary metal oxide semiconductor (CMOS) camera. Automated data collection was performed using Leginon (123). Particles were picked using DogPicker in the Appion software suite (124), extracted using Relion 3.0 (125), and imported into cryoSPARC v2 (126). After one round each of 2D and 3D classification, 14,027 particles were included in a 3D refinement with C3 symmetry imposed and a low-pass-filtered volume of ligand-free HIV-1 Env used as the initial model. The final resolution for the negative-stain reconstruction was estimated to be ∼25 Å (Fourier shell correlation cutoff of 0.5).

### Glycopeptide analysis by mass spectrometry.

Three 50-μg aliquots of each sample were denatured for 1 h in 50 mM Tris-HCl, pH 8.0, containing 6 M urea and 5 mM DTT. Next, Env proteins were reduced and alkylated by adding 20 mM iodoacetamide (IAA) and incubated for 1 h in the dark, followed by 1 h of incubation with 20 mM DTT to eliminate residual IAA. The alkylated Env proteins were buffer exchanged into 50 mM Tris-HCl, pH 8.0, using Vivaspin columns (3 kDa) and digested separately overnight using trypsin, chymotrypsin, or elastase (MS grade; Promega) at a ratio of 1:30 (wt/wt). The next day, the peptides were dried and extracted using C_18_ Zip-tip (Merck Millipore). The peptides were dried again, resuspended in 0.1% formic acid, and analyzed by nano-liquid chromatography–electrospray ionization mass spectrometry (nanoLC-ESI MS) with an Easy-nLC 1200 (Thermo Fisher Scientific) system coupled to a Fusion mass spectrometer (Thermo Fisher Scientific) using higher-energy collision-induced dissociation (HCD) fragmentation. Peptides were separated using an EasySpray PepMap rapid-separation liquid chromatography (RSLC) C_18_ column (75 μm by 75 cm). A trapping column (PepMap 100 C_18_; 3 μm, 75 μm by 2 cm) was used in line with the LC prior to separation with the analytical column. The LC conditions were the following: 275-min linear gradient consisting of 0 to 32% acetonitrile in 0.1% formic acid over 240 min followed by 35 min of 80% acetonitrile in 0.1% formic acid. The flow rate was set to 200 nl/min. The spray voltage was set to 2.7 kV, and the temperature of the heated capillary was set to 40°C. The ion transfer tube temperature was set to 275°C. The scan range was 400 to 1,600 *m/z*. The HCD collision energy was set to 50%, appropriate for the fragmentation of glycopeptide ions. Precursor and fragment detection was performed using an Orbitrap at the following resolution: MS1 = 100,000 and MS2 = 30,000. The automatic gain control (AGC) target for MS1 was 4e5 and for MS2 was 5e4, and injection time was as follows: MS1 = 50 ms and MS2 = 54 ms.

Glycopeptide fragmentation data were extracted from the raw file using Byonic (version 3.5) and Byologic software (version 3.5; Protein Metrics Inc.). The glycopeptide fragmentation data were evaluated manually for each glycopeptide; the peptide was scored as true positive when the correct b and y fragment ions were observed along with oxonium ions corresponding to the glycan identified. The MS data were searched using the Protein Metrics 305 N-glycan library. The relative amounts of each glycan at each site, as well as the unoccupied proportion, were determined by comparing the extracted chromatographic areas for different glycotypes with an identical peptide sequence. All charge states for a single glycopeptide were summed. The precursor mass tolerance was set to 4 parts per million (ppm) and 10 ppm for fragments. A 1% false-discovery rate (FDR) was applied. Glycans were categorized according to the composition detected. HexNAc(2)Hex(9–5) was classified as M9 to M5 and HexNAc(3)Hex(5–6)X as Hybrid with HexNAc(3)Fuc(1)X classified as Fhybrid. Complex-type glycans were classified according to the number of HexNAc residues, which are attributed to the number of processed antenna/bisecting GlcNAc (B) and fucosylation (F). For example, HexNAc(3)Hex(3-4)X was assigned to A1, HexNAc(4)X to A2/A1B, HexNAc(5)X to A3/A2B, and HexNAc(6)X to A4/A3B. If all of these compositions had a fucose, then they were assigned to the corresponding fucosylated antennary (FA) category. Note that this analytical approach does not distinguish between isomers, which could influence formal assignment of the number of antennae in some cases.

### Rabbit immunization and sample collection.

The Institutional Animal Care and Use Committee (IACUC) guidelines were followed for the animal subjects tested in the immunization studies. Rabbit immunization and blood sampling were performed under a subcontract at Covance (Denver, PA) following a previously described protocol (37). Three groups of female New Zealand White rabbits, four rabbits per group, were immunized intramuscularly with 30 μg of trimer or NP formulated in 250 μl of adjuvant AddaVax (InvivoGen) with a total volume of 500 μl, at weeks 0, 4, 12, 20, and 28. Blood samples (15 ml each) were collected at day −10 and weeks 1, 6, 14, 22, 28, and 30. Plasma was separated from blood and heat inactivated for the ELISA binding and TZM-bl neutralization assays.

### Data availability.

All data and codes to understand and assess the conclusions of this research are available in the main text, supplemental material, PDB (accession codes 7KLC, 7KKZ, and 7KMD), and EMDB (accession code EMD-22999). Additional data related to this paper may be requested from the authors.
