# Supplementary material for: Neutralizing Antibodies Induced by First-Generation gp41-Stabilized HIV-1 Envelope Trimers and Nanoparticles
Source: mBio. 2021 Jun 22;12(3):e00429-21. doi: 10.1128/mBio.00429-21 (PMC8262854; doi:10.1128/mBio.00429-21)
Supplement: FIG S1 [file mbio.00429-21-sf001.pdf]

Fig S1

A

Env-specific bulk sorting of mouse splenic B cells from the I3-01 nanoparticle group [a]

Mouse sample	Total sorted trimer-specific B cells	%Trimer-specific B cells
G5-1	578	0.06%
G5-2	87	0.01%
G5-3	727	0.01%
G5-4	1064	0.03%

[a] The sorting protocol and the bait are described in the Methods.

B

Next-generation sequencing (NGS) analysis of Env-specific splenic B cells from mice immunized with a 60-meric I3-01 nanoparticle presenting the HR1-stabilized BG505 trimer [a]

Mouse sample	N_{Raw}	N_{Align}	Chain	N_{Chain}	<Length>	N_{Usable}	$Perc_{Usable}$
G5-1	1,228,018	238,985	H	16,832	606.2	5,145	30.6%
			K	222,153	501.4	221,019	99.5%
G5-2	297,492	29,218	H	23,880	605.2	21,124	88.5%
			K	5,338	361.5	745	14.0%
G5-3	190,567	117,225	H	17,946	828.4	17,709	98.7%
			K	99,279	481.2	99,031	99.8%
G5-4	315,064	202,970	H	58,370	620.9	49,753	85.2%
			K	144,600	495.7	144,194	99.7%

[a] Listed items include the mouse sample ID, number of raw reads (N_{Raw}), number of sequences after V_H/V_K gene assignment and removing fragments with a V-gene alignment of 250bp or shorter (N_{Align}), Chain type (H or K), number of V_H/V_K chains, average read length of specific chain type, number of usable full-length antibody chains after the *Antibodyomics* pipeline processing (N_{Usable}), and percentage of usable chains ($Perc_{Usable}=N_{Usable}/N_{Chain}\times100\%$). NGS was performed on Ion S5 using an Ion 530 chip.

C

Amino acid sequences of consensus antibody chains identified from NGS analysis of Env-specific bulk-sorted splenic B cells [a]

Antibody chains from mouse 1 in the I3-01 group

>M1H1[b]	GAAGTTCAGCTATTGGAGACTGGAGGAGGCTTGGTGCAACCTGGGGGGTCACGGGGACTCTCTTGTGAAGGCTCAGGGTTTAGTTTTAGTGGCTTCTG<u>G</u>ATGAACTGGGTTCGACAGACACCTGGGAA<br>GACCCTGGACTGGATTGGAGACATTAATTCTGATGGCACATCAATAAGCTACGCACCCTCCATAAAGGATCGATTCACTGTCTTCAGATACACTGACAAGGACACCCTGTATCTGCAGATGAACAATG<br>TGCGATCTGAAGACACAGCCCCGTATTTCTGTATGAGGGGGTTTTACTTACTGGGGCCAAGGCTTACTGGGGCCAAGGGACTCTGGTCACTGTCTCTACA
>M1H2	GAAGTGAACCTTGAGGAGTCTGGAGGAGGCTTGGTGCAACCTGGAGGATCCATGAAACTCTCCTGTGTTGCCTCTGGATTCACTATCAGTAACTACTGGATGAACTGGGTCCGCCAGTCTCCAGAGAA<br>GGGGCTTGAGTGGGTTGCTGAAATTAGATTGAAAGCTAATAATGATGCAACACATTATGCGGAGTCTGTGAAAGGGAGGTTCACCATCTCAAGAGATGATTCCAAAAATAGTGTCTACCTGCAAATGA<br>ACAACTTAAGAGCTGAAGACACTGCCAATTATTACTGTACCAGGCCCGGTTACTACGGCTACTATGCTATGGACCAGTGGGGTCAAGGAACCTCAGTCACCGTCTCCTCA
>M1H3	CAGGTTCAGTTGCAGCAGTCTGGCGCTGAGTTGGTGAAACCTGGGGCCTCAGTGAAGATATCCTGCAAGGCTTCTGGCTACACCTTCACTGACCGTGCTATTCACTGGGTGAAACAGAAGCCTGAACA<br>GGGCCTGGAATGGATTGGATATATAGTTCCCGGAAATAGTGATATTAAGTACAGTGAGAAGTTCAAGGGCAAGGCCACACTGACTGCAGACAAATCCTCCACCACTGCCTACATGCAGGTCAACAGCC<br>TGACATCTGAGGACTCTGCAGTGTATTTCTGTAATTGCTATGATTACGACGACGGCTATTGGGGTCAAGGAACCTCAGTCACCGTCTCCTCA
>M1K1	GACATTGTGCTGACCCAATCTCCAGCTTCTTTGGCTGTGTCTCTAGGACAGAGGGCCACCATCTCCTGCAGAGCCAGCGAAAGTGTTGATAATTATGGCATTAGTTTTATGAACTGGTTCCAACAGAA<br>ACCAGGACAGCCACCCAAACTCCTCATCTATGGTGCATCCAACCAAGGATCCGGGGTCCCTGCCAGGTTTAGTGGCAGTGGGTCTGGGACGGATTTCAGCCTCAACATCCATCCTATGGAGGAGGATG<br>ATACTGCAATGTTTTTCTGTCAGCAAAGTAAGGAGGTTCCGTACACGTTCGGAGGGGGGACCAAGGTGGAAATAAAA
>M1K2	GACATTGTGATGACCCAGTCTCACAAATTCATGTCCACATCAGTAGGAGACAGGGTCAGCATCACCTGCAAGGCCAGTCAGGATGTGAGTACTGCTGTAGCCTGGTATCAACAAAAACCAGGGCAATC<br>TCCTAAACTACTGTTTTACTGGACATCCGCCCGGCACACTGGAGTCCCTGATCGCTTCACAGGCAGTGGATCTGGGACAGATTATACTCTCACCATCAACAATGTAATGGCTGAAGACCTGGCACTTT<br>ATTACTGTCAACAACATTATAGCACTCCGTGGACGTTCGGTGGAGGCACCAAGCTGGAGATCAAA
>M1K3	CAAATTGTTCTCACCCAGTCTCCACGAATCATGTCTGCATCTCCAGGGGAGAGGGTCACCGTGACCTGCAGTGCCAGCTCAAGTGTAAGTTACATGTCCTGGTACCAGCAGAAGCCAGGATCCTCCCC<br>CAGACTCCTGATTTTTGACACATCCAACCTGGCTTCTGGAGTCCCTTTTCGCTTCAGTGGCAGTGGGTCTGGGACCTCTTACTCTCTCACAATCAGCCGAATGGAGGCTGAAGATGCTGCCACTTATT<br>GCTGCCAGCAGTGGAGTCGTTACCCATTCACGTTCGGCTCGGGGACAAAGTTGGAAATAAAA

Antibody chains from mouse #4 in the I3-01 group

>M4H1[b]	CAGGTCCAGTTGCAGCAGTCTGGAGCTGACCTGGTCAGGCCTGGGACTTCATTGAAGAAGTCCTCCAAGGTTTCTGGCTACACCTTCACTAACTACTGGATAGGTTGGGTAAAGCAGAGGCCTGGACA<br>TGGCCTTGAGTGGATTGGAGATGTTTACCCTGGAGACGGTTTTACTCAGAACAATGAGAAGTTCAAGGACAAGGCCACACTGACTGCAGACAAATCCTCCAGCACATCCTACAGG<u>C</u>A<u>G</u>CTCAGCAGTC<br>TGACATCTGAGGAATCTGCGGTCCATTA<u>C</u>TGTTCGACACCTACGGTAGTGCCTGACTACTGGGGCCAAGGCACCACTCTCACAGTCTCCTCA
>M4H2	GAAGTGAAGCTTGAGGAGTCCGGAGGAGGCTTGGTGCAACCTGGAGGATCCATGAAACTCTCCTGTGTTGCCTCTGGAATCACTTTCAGTAACTCCTGGATGAGCTGGGTCCGCCAGTCTCCAGAGAA<br>GGGGCTTGAGTGGGTTGCTGAAATTAGATTGAAAGCTCAAAATTATGCAACACATTATGCGGCGTCTGTGAAAGGGAGGTTCACCATCTCAAGAGATGACTCCAAAAGTAGTGTCTACCTACAAATGA<br>ACAACTTAAGACCTGAAGACACTGGCATCTATTACTGTACCACCCCACTGGGAGGCTATTTTGATATGGACTACTGGGGTCAAGGAACCTCACTCACCGTCTCCTCA
>M4K1	GACATTGTGCTGACCCAATCTCCAGCTTCTTTGGCTGTGTCTCTAGGGCAGAGGGCCACCATCTCCTGCAGAGCCAGCGAAAGTGTTGATATTTATGGCATTAGTTTTATGAACTGGTTCCAACAGAG<br>ACCAGGACAGCCACCCAAACTCCTCATCTATGCTGCATCCAACCGAGGATCCGGGGTCCCTGCCAGGTTTAGTGGCAGTGGGTCTGGGACAGACTTCAGCCTCAACATCCATCCTATGGAGGAGGATG<br>ATACTGCAATGTATTTCTGTCAGCAAAGTAAGGAGGTTCCGTGGACGTTCGGTGGAGGCACCAAGCTGGAAATCAAA
>M4K2	CAAATTGTTCTCACCCAGTCTCCAGCAATCATGTCTGCATCTCCAGGGGAGAAGGTCACCATGACCTGCAGTGCCAGCTCAAGTGTAAGTTACATGTCTTGGTACCAGCAGAAGCCAGGATCCTCCCC<br>CCGACTCCTGATTTATGACACATCCGACCTGGCTTCTGGAGTCCCTTTTCGCTTCAGTGGCAGTGGGTCTGGGACCTCTTACTCTCTCACAATCAGCCGAATGGAGGCTGAAGATGCTGCCACTTATT<br>ACTGCCAACAGTGGGATCCTTACCCGCTCACGTTCGGTTCTGGGACCAAGCTGGAGCTGAAA
>M4K3	GACATTGTGATGTCACAGTCTCCATCCTCCCTAGCTGTGTCAGTTGGAGAGAAGGTTACTATGAGCTGCAAGTCCAGTCAGAGCCTTTTATATAGTAGCAATCAAAAGAACTACTTGGCCTGGTACCA<br>GCAGAAACCAGGGCAGTCTCCTAAACTGCTGATTTACTGGGCATCCACTAGGGAATCTGGGGTCCCTGATCGCTTCACAGGCAGTGGATCTGGGACAGATTTCACTCTCACCATCAGCAGTGTGAAGG<br>CTGAAGACCTGGCAGTTTATTACTGTCAGCAATATTATAGCTATCCGCTCACGTTCGGTGCTGGGACCAAGCTGGAGCTGAAA

[a] The antibody chain sequences were identified from the NGS of Env-specific splenic B cells using a clustering algorithm and consensus as described in detail in Methods.
[b] Nucleotide that was modified to remove a stop codon is colored in red and underscored.

Amino acid sequences of consensus antibody chains identified from NGS analysis of Env-specific bulk-sorted splenic B cells [a]

Antibody chains from mouse 1 in the I3-01 group

>M1H1[b]	EVQLLETGGGLVQPGGSRGLSCEGSGFSFSGFWMNWVRQTPGKTLDWIGDINSDGTSISYAPSIKDRFTVFRYTDKDTLYLQMNNVRSEDTAPYFCMRGFYLLGPRLTGAKGLWSLSL
>M1H2	EVNLEESGGGLVQPGGSMKLSCVASGFTISNYWMNWVRQSPEKGLEWVAEIRLKANNDATHYAESVKGRFTISRDDSKNSVYLQMNNLRAEDTANYYCTRPGYYGYYAMDQWGQGTSVTVSS
>M1H3	QVQLQQSGAELVKPGASVKISCKASGYTFTDRAIHWVKQKPEQGLEWIGYIVPGNSDIKYSEKFKGKATLTADKSSTTAYMQVNSLTSEDSAVYFCNCYDYDDGYWGQGTSVTVSS
>M1K1	DIVLTQSPASLAVSLGQRATISCRASESVDNYGISFMNWFQQKPGQPPKLLIYGASNQGSGVPARFSGSGSGTDFSLNIHPMEEDDTAMFFCQQSKEVPYTFGGGTKVEIK
>M1K2	DIVMTQSHKFMSTSVGDRVSITCKASQDVSTAVAWYQQKPGQSPKLLFYWTSARHTGVPDRFTGSGSGTDYTLTINNVMAEDLALYYCQQHYSTPWTFGGGTKLEIK
>M1K3	QIVLTQSPRIMSASPGERVTVTCSASSSVSYMSWYQQKPGSSPRLLIFDTSNLASGVPFRFSGSGSGTSYSLTISRMEAEDAATYCCQQWSRYPFTFGSGTKLEIK

Antibody chains from mouse 4 in the I3-01 group

>M4H1	QVQLQQSGADLVRPGTSLKKSSKVSGYTFTNYWIGWVKQRPGHGLEWIGDVYPGDGFTQNNEKFKDKATLTADKSSSTSYRQLSSLTSEESAVHYCSTPTVVPDYWGQGTTLTVSS
>M4H2	EVKLEESGGGLVQPGGSMKLSCVASGITFSNSWMSWVRQSPEKGLEWVAEIRLKAQNYATHYAASVKGRFTISRDDSKSSVYLQMNNLRPEDTGIYYCTTPLGGYFDMDYWGQGTSLTVSS
>M4K1	DIVLTQSPASLAVSLGQRATISCRASESVDIYGISFMNWFQQRPGQPPKLLIYAASNRGSGVPARFSGSGSGTDFSLNIHPMEEDDTAMYFCQQSKEVPWTFGGGTKLEIK
>M4K2	QIVLTQSPAIMSASPGEKVTMTCSASSSVSYMSWYQQKPGSSPRLLIYDTSDLASGVPFRFSGSGSGTSYSLTISRMEAEDAATYYCQQWDPYPLTFGSGTKLELK
>M4K3	DIVMSQSPSSLAVSVGEKVTMSCKSSQSLLYSSNQKNYLAWYQQKPGQSPKLLIYWASTRESGVPDRFTGSGSGTDFTLTISSVKAEDLAVYYCQQYYSYPLTFGAGTKLELK

[a] The amino acid sequences were translated from the nucleotide sequences using TRANSEQ.
[b] HCDR3 and framework 4 (FR4) region missing the “WGXG” motif and “VSS” motifs are highlighted in gray shading.

D

Nucleotide sequences of two antibodies identified from splenic B cells of mouse 4 (M4) in the I3-01 group by Env-specific single-cell sorting [a]

```
>M4-Ab3H CAGGTGCAGCTGCAGCAGCCTGGAGGAGGCTCGGTGCAACCTGGAGGATCCATGAAACTCTCCTGTGTTGCCTCTGGATTCACCTTCAGTAATTCCTGGATGAACTGGGTCCGCCAGTCTCCAGAGAA
         GGGGCTTGAGTGGGTTGCTGAAATTCGATTGAAAGTTCATAATTATGCAACACATTATGCGGAGTCTGTGAAAGGGAGGTTCACCATCTCAAGAGATGATTCCAAAAGTAGTGTCTACCTGCAAATGA
         TCAACTTAAGACCAGAAGACACTGGCATTTATTATTGTACTACCCCACTGGGTGGCTACTTTCCTATGGACTACTGGGGTCAAGGAACCACTCTCACAGTCTCCTCA
>M4-Ab3K GACATTGTGCTGACCCAATCTCCAACTTCTTTGGCTGTGTCTCTAGGGCAGAGGGCCACCATCTCCTGCAGAGCCAGCGAAAGTGTTGATAATTATGGCGTTAGTTTTATGAACTGGTTCCAACAGAA
         ACCAGGACGGCCACCCAAACTCCTCATCTATGCTGCATCCAAGCAAGGATCCGGGGTCCCTGCCAGGTTTAGTGGCAGTGGGTCTGGGACAGATTTCAGCCTCAACATCCATCCAATGGAGGAGGATG
         ATATTGCAATGTATTTCTGTCAGCAAAATAAGGAGCTTCCGTGGACGTTCGGTGGAGGCACCAAGCTGGAAATCAAA
>M4-Ab9H AGGGTGCAGCTGCAGCAGTCTTGTGGAGGCTTGGTGCAACCTGGAGGATCCATGAAACTCTCCTGCGTTGCCTCTGGAATCACTTTCAGTAACTCCTGGATGAACTGGGTCCGCCAGTCTCCAGAGAA
         GGGGCTTGAGTGGGTTGCTGAAATTAGATTGAAAGTTAATAATTATGCAACACATTATGCGGAGTCTGTGAAAGGGAGGTTCACCATCTCAAGAGATGATTCCAAAAGGAGTGTCTACCTGCAAATGA
         ACAACTTAAGAGCTGAAGACACTGGCATTTATTACTGTACCACCCCACTGGGTGGCTACTATGCTGTGGACTACTGGGGTCAAGGAGCCACTCTCACAGTCTCCTCA
>M4-Ab9K GACATCCAGATGATTCAGTCTCCAGCTTCTTTGGCTGTGTCTCTAGGGCAGAGGGCCACCATCTCCTGCAGAGCCAGCGAAAGTGTTGATAATTATGGCATTAGTTTTATGAACTGGTTCCAACAGAA
         ACCAGGACAGCCACCCAAACTCCTCATCTATGCTGCATCCAACCAAGGATCCGGGGTCCCTGCCAGGTTTAGTGGCAGTGGGTCTGGGACAGACTTCAGCCTCAACATCCATCCTATGGAGGAGGATG
         ATACTGCAATGTATTTCTGTCAGCAAAGTAAGGAGGTTCCGTGGACGTTCGGTGGAGGCACCAAGCTGGAAATCAAA
```

[a] The antibody chain sequences were identified from Env-specific single-cell sorting, PCR and cloning as described in Methods.

Amino acid sequences of two antibodies identified from splenic B cells of mouse 4 (M4) in the I3-01 group by Env-specific single-cell sorting [a]

```
>M4-Ab3H    QVQLQQPGGGSVQPGGSMKLSCVASGFTFSNSWMNWVRQSPEKGLEWVAEIRLKVHNYATHYAESVKGRFTISRDDSKSSVYLQMINLRPEDTGIYYCTTPLGGYFPMDYWGQGTTLTVSS
>M4-Ab3K    DIVLTQSPTSLAVSLGQRATISCRASESVDNYGVSFMNWFQQKPGRPPKLLIYAASKQGSGVPARFSGSGSGTDFSLNIHPMEEDDIAMYFCQQNKELPWTFGGGTKLEIK
>M4-Ab9H    RVQLQQSCGGLVQPGGSMKLSCVASGITFSNSWMNWVRQSPEKGLEWVAEIRLKVNNYATHYAESVKGRFTISRDDSKRSVYLQMNNLRAEDTGIYYCTTPLGGYYAVDYWGQGATLTVSS
>M4-Ab9K    DIQMIQSPASLAVSLGQRATISCRASESVDNYGISFMNWFQQKPGQPPKLLIYAASNQGSGVPARFSGSGSGTDFSLNIHPMEEDDTAMYFCQQSKEVPWTFGGGTKLEIK
```

[a] The amino acid sequences were translated from the nucleotide sequences using TRANSEQ.

Fig S1 HIV-1 Env-specific sorting and NGS of mouse splenic B cells for antibody isolation. Mice immunized with BG505 gp140.664.R1-PADRE-I3-01 nanoparticle (see ref. 37) were analyzed in this study. (**A**) Env-specific mouse splenic B cells obtained from bulk sorting using a biotinylated Avi-tagged BG505 gp140.664.R1 trimer probe. (**B**) Antibodyomics pipeline processing of NGS data obtained from sequencing of Env-specific mouse splenic B cells on the Ion S5 platform. **(C)** Nucleotide and amino acid sequences of consensus antibody heavy and κ-light chains (HC and KC) identified from NGS analysis of Env-specific splenic B cells from mice 1 and 4 (M1 and M4) in the I3-01 nanoparticle group. (**D**) Nucleotide and amino acid sequences of two antibodies, Ab3 and Ab9, identified by single-cell sorting and antibody cloning from M4 splenic B cells. Ab3 and Ab9 use the same germline genes as the NGS-derived NAb, M4H2K1.
