# Supplementary material for: Neutralizing Antibodies Induced by First-Generation gp41-Stabilized HIV-1 Envelope Trimers and Nanoparticles
Source: mBio. 2021 Jun 22;12(3):e00429-21. doi: 10.1128/mBio.00429-21 (PMC8262854; doi:10.1128/mBio.00429-21)
Supplement: FIG S2 [file mbio.00429-21-sf002.pdf]

Fig S2

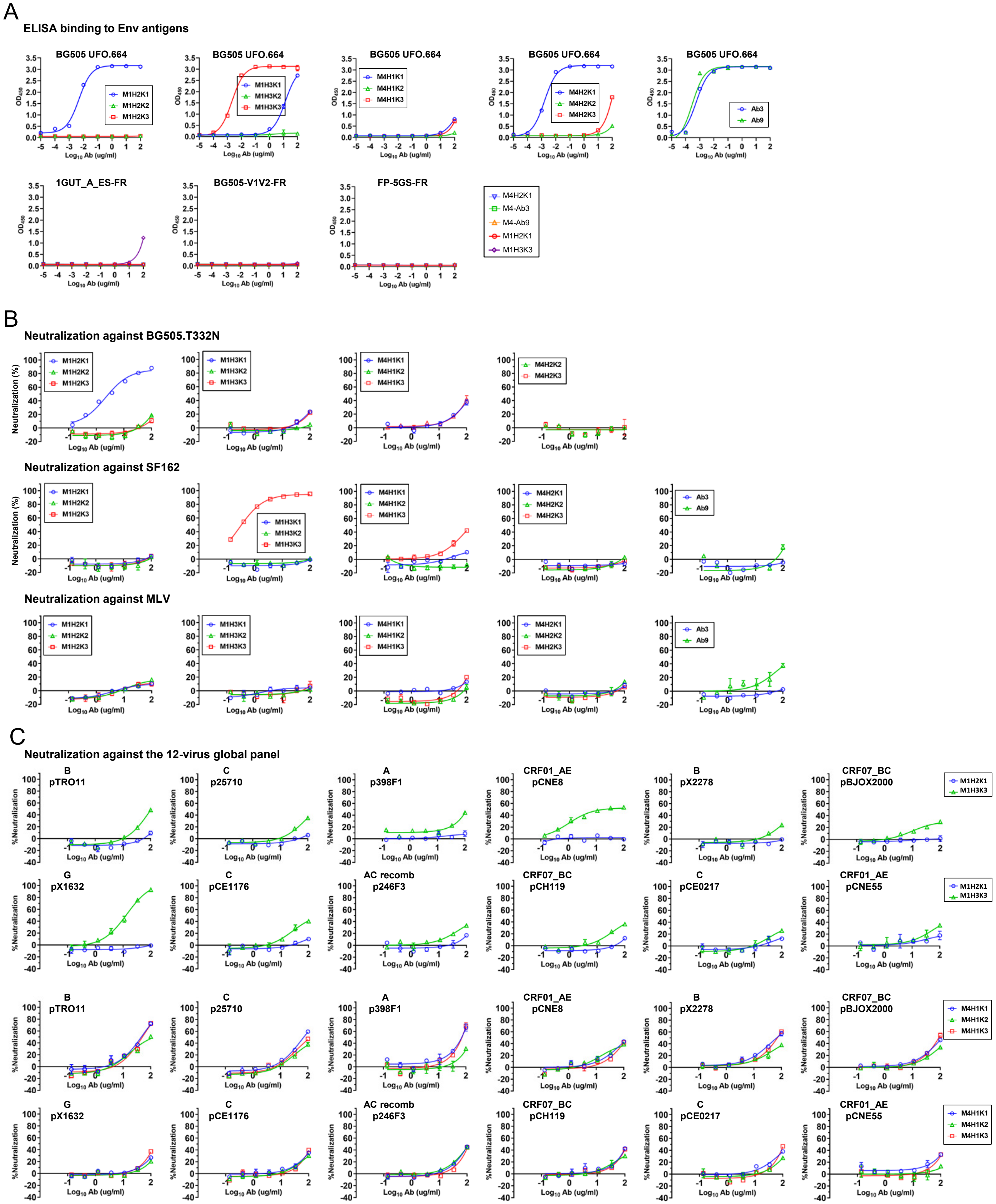
A
ELISA binding to Env antigens
BG505 UFO.664
M1H2K1
M1H2K2
M1H2K3
M1H3K1
M1H3K2
M1H3K3
M4H1K1
M4H1K2
M4H1K3
M4H2K1
M4H2K2
M4H2K3
Ab3
Ab9
1GUT_A_ES-FR
BG505-V1V2-FR
FP-5GS-FR
M4-Ab3
M4-Ab9
OD450
Log10 Ab (ug/ml)
B
Neutralization against BG505.T332N
Neutralization against SF162
Neutralization against MLV
Neutralization (%)
C
Neutralization against the 12-virus global panel
B pTRO11
C p25710
A p398F1
CRF01_AE pCNE8
B pX2278
CRF07_BC pBJOX2000
G pX1632
C pCE1176
AC recomb p246F3
CRF07_BC pCH119
C pCE0217
CRF01_AE pCNE55
%Neutralization

C (continued)

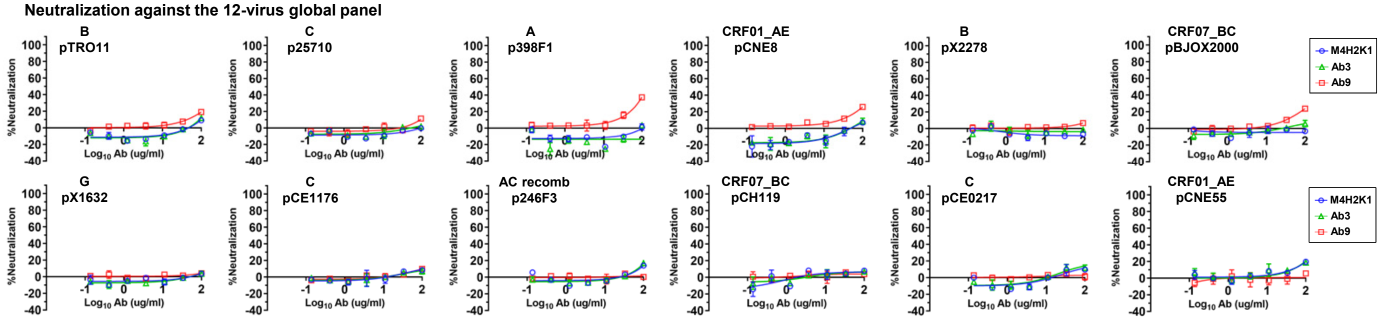

Fig S2 Functional evaluation of NGS and single-cell-derived mouse mAbs. (**A**) ELISA binding of mouse mAbs to Env antigens including BG505 UFO.664 trimer (top panel) and three individual epitope probes including 1GUT_A_ES-FR (N332 supersite), BG505 V1V2-FR (V1V2 apex), and FP-5GS-FR (fusion peptide), which are all ferritin nanoparticles. **(B)** Neutralization of autologous tier 2 clade A BG505.T332N by mouse mAbs. **(C)** Neutralization of all 12 isolates from a global panel by mouse mAbs.
