# Supplementary material for: Neutralizing Antibodies Induced by First-Generation gp41-Stabilized HIV-1 Envelope Trimers and Nanoparticles
Source: mBio. 2021 Jun 22;12(3):e00429-21. doi: 10.1128/mBio.00429-21 (PMC8262854; doi:10.1128/mBio.00429-21)
Supplement: FIG S3 [file mbio.00429-21-sf003.pdf]

Fig S3

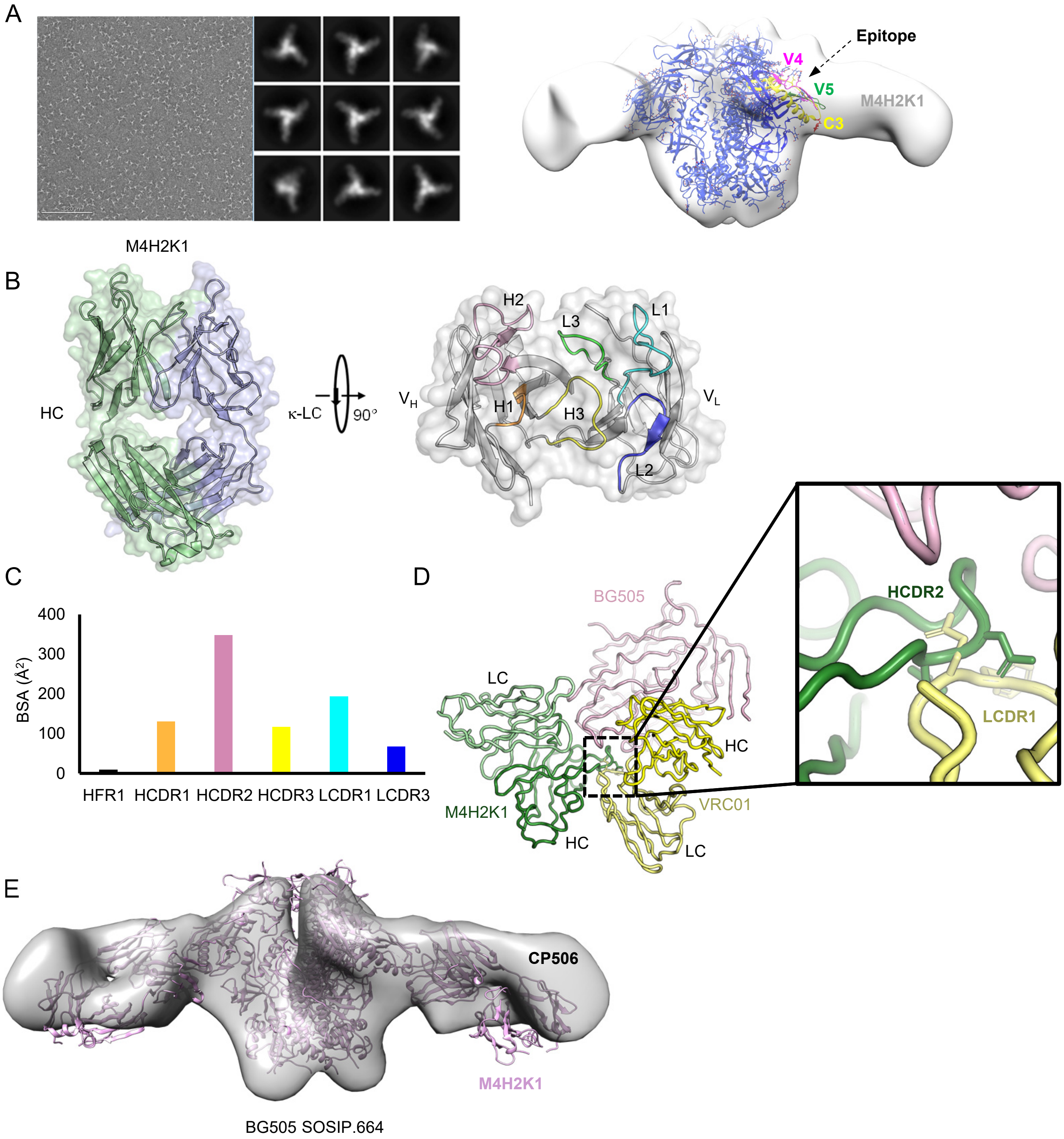

Fig S3 Structural characterization of the NGS-derived mouse NAb, M4H2K1. (**A**) Negative-stain EM (nsEM) analysis of mouse NAb M4H2K1 in complex with BG505 UFO.664 Env trimer. Left: EM micrograph and 2D class averages. Right: Side view of BG505 Env (PDB ID: 4ZMJ) docked into 3D reconstruction, with the potential epitope color-coded on the Env trimer structure (C3: yellow; V4: magenta; V5: green). Glycan moieties are shown as sticks. (**B**) The unbound structure of M4H2K1 in a ribbons model within the molecular surface. Left: side view; Right: top view. The H/LCDR loops are labeled on the structure. (**C**) Buried surface area (Å^2) of the CDR loops and FRs of M4H2K1 Fab when bound to BG505 gp120 core. (**D**). Superimposition of VRC01 (yellow) Fab-bound BG505 SOSIP with M4H2K1 (green) Fab-bound BG505 core (pink). The right inset shows a clash of M4H2K1 HCDR2 with VRC01 LCDR1. (**E**) Comparison of the mode of recognition for M4H2K1 and CP506 when bound to the BG505 SOSIP.664 Env trimer.
