# Supplementary material for: Neutralizing Antibodies Induced by First-Generation gp41-Stabilized HIV-1 Envelope Trimers and Nanoparticles
Source: mBio. 2021 Jun 22;12(3):e00429-21. doi: 10.1128/mBio.00429-21 (PMC8262854; doi:10.1128/mBio.00429-21)
Supplement: FIG S4 [file mbio.00429-21-sf004.pdf]

Fig S4

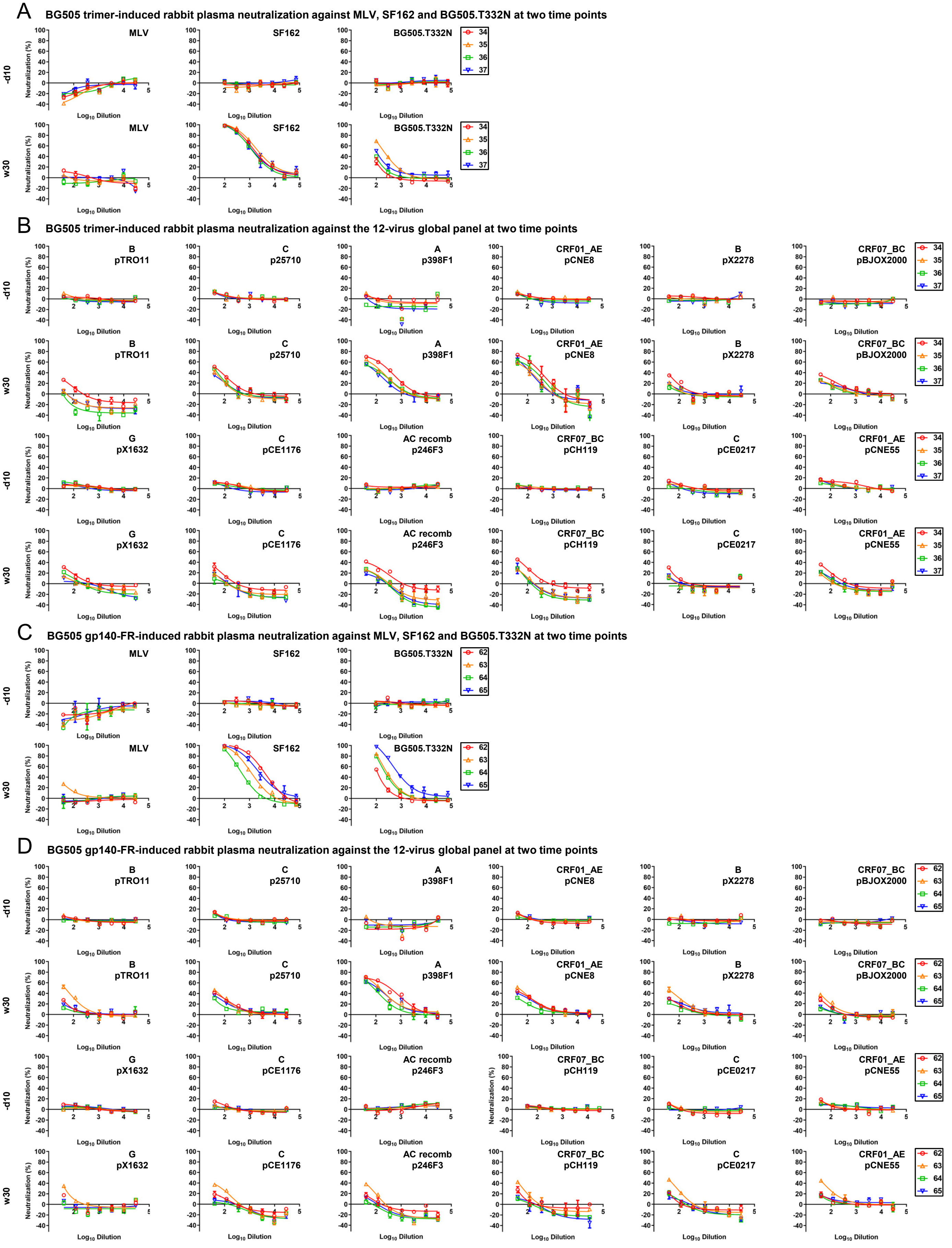
A
BG505 trimer-induced rabbit plasma neutralization against MLV, SF162 and BG505.T332N at two time points
-d10
w30
MLV
SF162
BG505.T332N
34
35
36
37
Neutralization (%)
Log10 Dilution
B
BG505 trimer-induced rabbit plasma neutralization against the 12-virus global panel at two time points
B pTRO11
C p25710
A p398F1
CRF01_AE pCNE8
B pX2278
CRF07_BC pBJOX2000
G pX1632
C pCE1176
AC recomb p246F3
CRF07_BC pCH119
C pCE0217
CRF01_AE pCNE55
C
BG505 gp140-FR-induced rabbit plasma neutralization against MLV, SF162 and BG505.T332N at two time points
62
63
64
65
D
BG505 gp140-FR-induced rabbit plasma neutralization against the 12-virus global panel at two time points

Fig S4 Rabbit plasma neutralization from two BG505 Env-immunized rabbit groups. In the previous study (see ref. 37), two groups of rabbits were immunized with BG505 gp140.664.R1 trimer and its ferritin nanoparticle. (**A**) Neutralization of MLV, tier 1 clade B SF162, and tier 2 clade A BG505.T332N by day -10 (-d10) and week 30 (w30) rabbit plasma from the BG505 trimer group. (**B**) Neutralization of 12 isolates in the global panel by day -10 (-d10) and week 30 (w30) rabbit plasma from the BG505 trimer group. (**C**) Neutralization of MLV, SF162, and BG505.T332N by day -10 (-d10) and week 30 (w30) rabbit plasma from the BG505 ferritin nanoparticle group. (**D**) Neutralization of 12 isolates in the global panel by day -10 (-d10) and week 30 (w30) rabbit plasma from the BG505 ferritin nanoparticle group. The heat-inactivated rabbit plasma was diluted 100-fold for autologous tier 2 BG505.T332N and tier 1 SF162 and subjected to a 3-fold dilution series in the TZM-bl assay. To increase the sensitivity of detection, heat-inactivated plasma was diluted 40-fold for MLV and all 12 isolates from a global panel and followed by a 3-fold dilution series in the TZM-bl assays. ID_{50} titers for plots in (A) – (D) are shown in Fig 3B.
