# Supplementary material for: Neutralizing Antibodies Induced by First-Generation gp41-Stabilized HIV-1 Envelope Trimers and Nanoparticles
Source: mBio. 2021 Jun 22;12(3):e00429-21. doi: 10.1128/mBio.00429-21 (PMC8262854; doi:10.1128/mBio.00429-21)
Supplement: FIG S5 [file mbio.00429-21-sf005.pdf]

Fig S5

A Antibody isolation by single B-cell sorting, cloning and screening

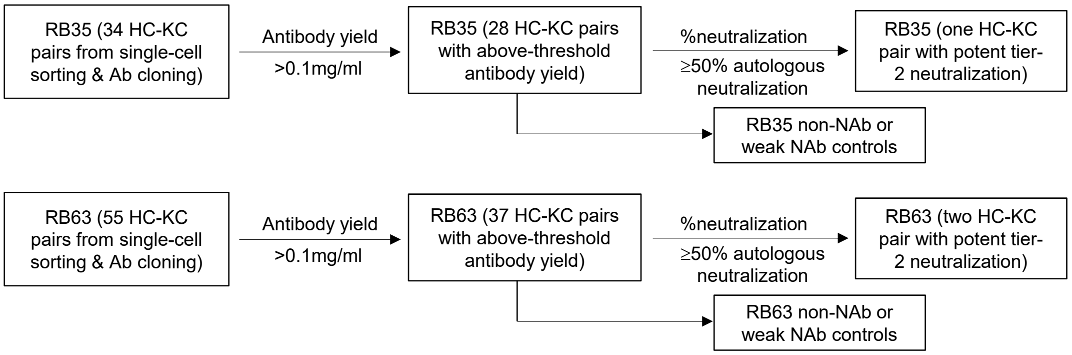

B Sequences of three rabbit monoclonal antibodies (mAbs)

```
>RB35-1B11 HC (IGHV1S45*01/IGHD6-1*01/IGHJ4*01)
QLEESGGGLVKPGGTLTLTCKASGFDFYDGVYMCWVRQAPGKGLEWIGCIFTDNSRTYYASWAKGRFTISKTSSTTVTLQMTSLTAADTATYFCTRDYPGDADPYRLWGPGTLVTVSS
>RB35-1B11 KC (IGKV1S36*01/IGKJ1-2*01)
DIVMTQTPASVSAAVGGTVTIKCQASESIYSNLAWYQQKPGQAPKVLIYGSSNLESGVPSRFKGSGSGAEYTLTISDLECADAATYYCQCTYDVTTGGYYGNSFGGGTGVLVK

>RB63-1E7 HC (IGHV1S40*01/IGHD2-1*01/IGHJ4*01)
HSQLVESGGGLVQPGASLTLTCKASGFSFSDGYYISWVRQAPGKGLEWIGWIYTDSGVTSYASWAHGRFTISKTSSTTVTLQMTSLTAADTATYFCARVDHDRDYRAVRGKLWGPGTLVTVSS
>RB63-1E7 KC (IGKV1S10*01/IGKJ1-2*01)
ELVMTQTPASVEAAVGGTVTIKCQASQSISNYLSWYQQKPGQPPKLLIYRASTLESGVPSRFKGSGSGTQFTLTISDLECADAATYYCQCTFGTAVDRGFGDTFGGGTEVVVK

>RB63-4B5 HC (IGHV1S40*01/undetermined IGHD/IGHJ2*01)
SQSLEESGGGLVQPGGSLTVTCKASGFSFSSSYWVCWVRQAPGKGLEWIGCIYNDYGHTAYASWVNGRFTISKPSSTTVTLQMTSLTAADTATYFCARGIELDWLNAFDPWGPGTLVTVSS
>RB63-4B5 KC (IGKV1S15*01/IGKJ1-2*01)
ELDMTQTPSSTSAAVGGTVTITCQSSESVWRNNWLAWYQQKTGQPPKLLIYLASTLASGVPSRFKGSGSGTQFTLTISGVQCEDAATYYCQGTYSSHAWYVTFGGGTEVVVK
```

C ELISA binding to three epitope probes

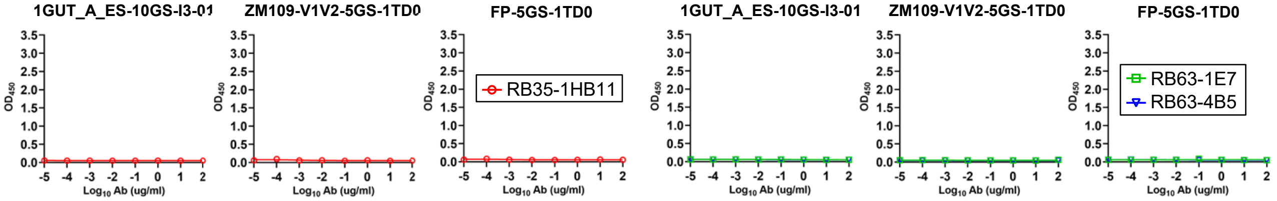

D Neutralization against a negative control, MLV

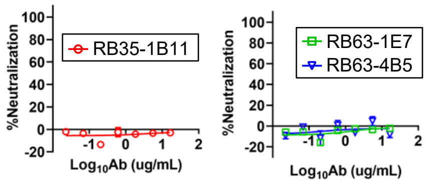

Fig S5

E

Neutralization against the 12-virus global panel

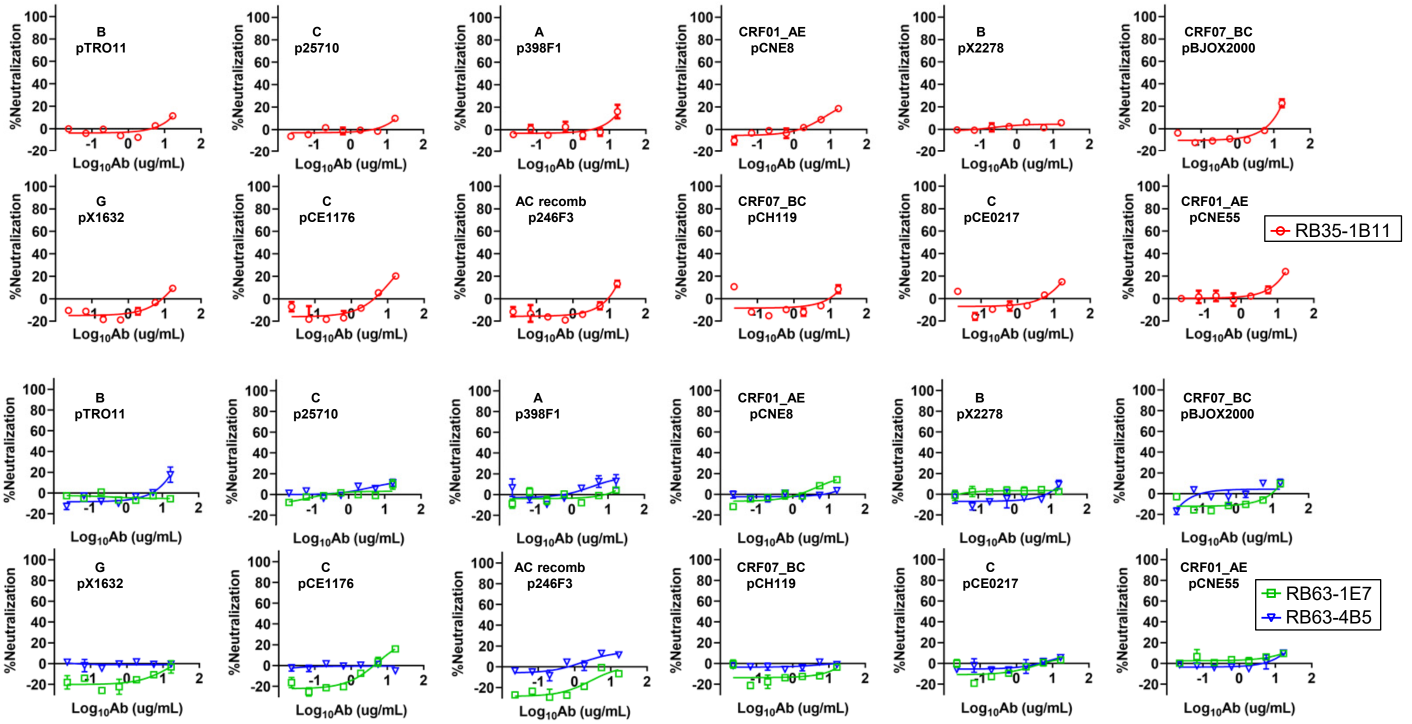

F

Neutralization against BG505.T332N

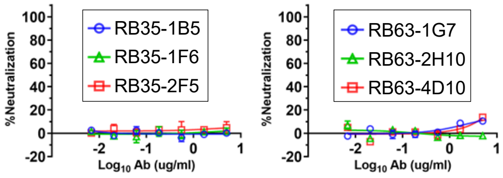

Fig S5 Functional evaluation of single-cell sorted rabbit mAbs. (**A**) Schematic representation of the procedure used to select functional mAbs from a rabbit immunized with BG505 gp140.664.R1 trimer (RB35) and a rabbit immunized with BG505 gp140.664.R1-FR nanoparticle (RB63). The two major selection criteria are: (1) yield ≥ 0.1mg/ml after purification and concentration, and (2) %neutralization ≥ 50% at 10ug/ml for BG505.T332N. Weak/non-NAbs matching only the first criterion may be selected for comparison. (**B**) Amino acid sequences of three rabbit NAbs identified from this screening procedure. (**C**) ELISA binding by the RB35/RB63 NAbs to an I3-01 nanoparticle presenting 24 copies of an N332 scaffold (1GUT_A_ES), a trimeric scaffold (1TD0) presenting ZM109 V1V2, and the same trimeric scaffold (1TD0) presenting fusion peptide (FP-5GS-1TD0). Antibodies were diluted to 100ug/ml and subjected to a 10-fold dilution series in the assay. (**D**) Neutralization of MLV by the RB35/RB63 NAbs. **(E)** Neutralization of all 12 isolates from a global panel by the RB35/RB63 NAbs. Antibodies were diluted to 33.3ug/ml and followed by a 3-fold dilution series in the TZM-bl assay. **(F)** ELISA binding of six non-NAbs, two from each rabbit, to BG505 UFO.664 trimer.
