# Supplementary material for: Neutralizing Antibodies Induced by First-Generation gp41-Stabilized HIV-1 Envelope Trimers and Nanoparticles
Source: mBio. 2021 Jun 22;12(3):e00429-21. doi: 10.1128/mBio.00429-21 (PMC8262854; doi:10.1128/mBio.00429-21)
Supplement: FIG S6 [file mbio.00429-21-sf006.pdf]

Fig S6

A

Env-specific bulk sorting of rabbit B cells from PBMCs of RB35 and RB63 [a]

Mouse sample	Total sorted trimer-specific B cells	%Trimer-specific B cells
RB35	363	0.01%
RB63	370	0.03%

[a] The sorting protocol and the bait are described in the Methods.

B

Next-generation sequencing (NGS) analysis of Env-specific B cells from two rabbits immunized with an HR1-stabilized BG505 trimer (RB35) and with a ferritin nanoparticle presenting 8 HR1-stabilized BG505 trimers[a]

Rabbit sample	N_{Raw}	N_{Align}	Chain	N_{Chain}	<Length>	N_{Usable}	$Perc_{Usable}$
RB35	1,116,425	628,914	H	243,116	601.3	179,476	73.8%
			K	385,712	525.6	240,635	62.4%
RB63	1,887,035	716,716	H	262,463	497.0	118,422	45.1%
			K	453,975	506.6	265,203	58.4%

[a] Listed items include the mouse sample ID, number of raw reads (N_{Raw}), number of sequences after V_H/V_K gene assignment and removing fragments with a V-gene alignment of 250bp or shorter (N_{Align}), Chain type (H or K), number of V_H/V_K chains, average read length of specific chain type, number of usable full-length antibody chains after the *Antibodyomics* pipeline processing (N_{Usable}), and percentage of usable chains ($Perc_{Usable}$=N_{Usable}/N_{Chain}×100%). NGS was performed on Ion S5 using an Ion 530 chip.

C

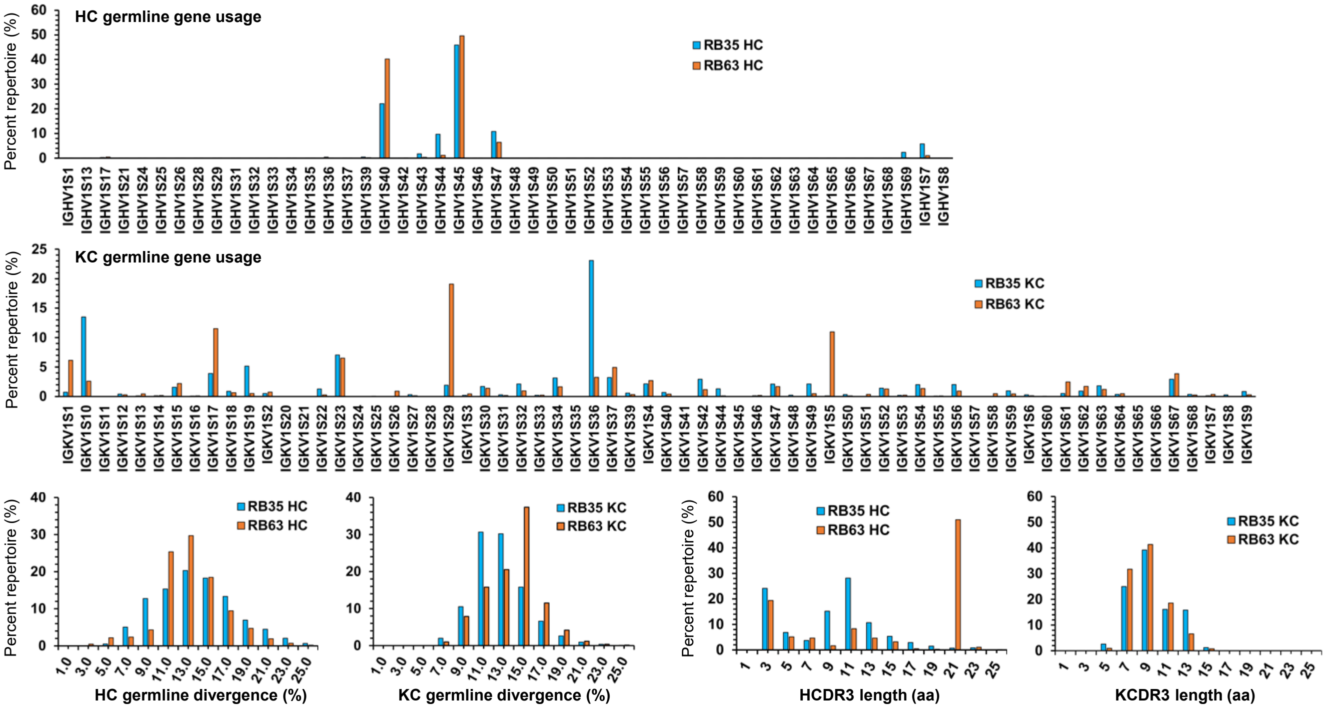

D

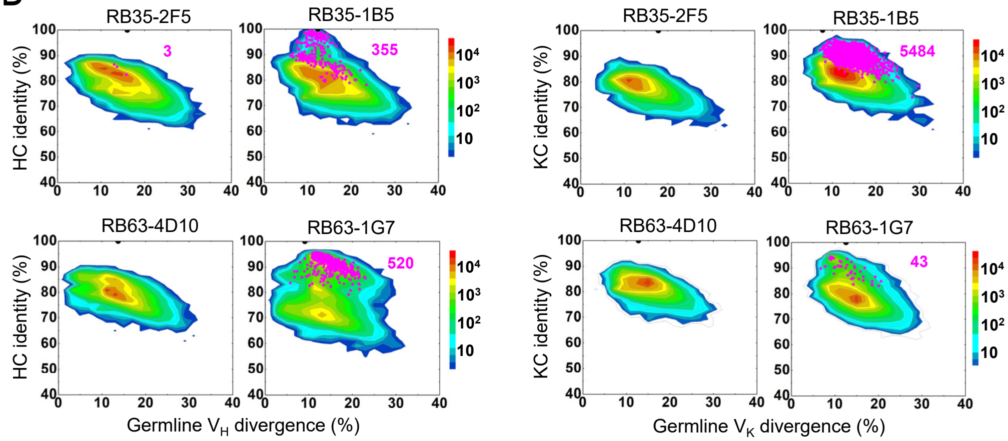

E BG505 trimer/gp140-FR-induced rabbit plasma neutralization against BG505.T332N and three glycan hole mutants

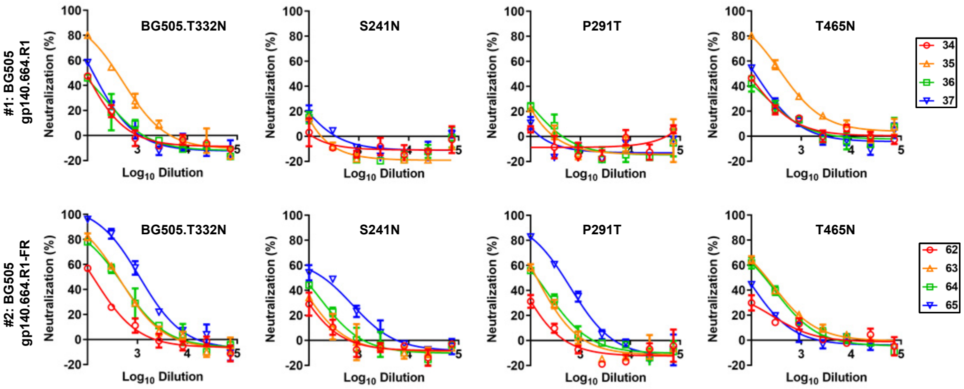

Fig S6 HIV-1 Env-specific sorting and NGS of rabbit B cells for antibody isolation. PBMCs from a rabbit immunized with BG505 gp140.664.R1 trimer (RB35) and a rabbit immunized with BG505 gp140.664.R1-FR10 nanoparticle (RB63) were analyzed. (**A**) Env-specific rabbit B cells obtained from bulk sorting using a biotinylated Avi-tagged BG505 gp140.664.R1 trimer probe. (**B**) Antibodyomics pipeline processing of NGS data obtained from sequencing of Env-specific rabbit B cells on the Ion S5 platform. **(C)** Quantitative B cell repertoire profiles derived from the NGS analysis of Env-specific RB35 and RB63 B cells, including HC and KC germline gene usage, somatic hypermutation (SHM), and CDR3 length. **(D)** Divergence-identity analysis of four representative non-NAbs in the context of Env-specific antibody repertories for RB35 and RB63. HC and KC sequences are plotted as a function of sequence identity to the template and sequence divergence from putative germline genes. Color coding indicates sequence density. Templates and sequences identified based on the CDR3 identity of 95% or greater are shown as black and magenta dots on the plots, respectively, with the number of sequences labeled accordingly. **(E)** Rabbit plasma neutralization from two BG505 Env-immunized rabbit groups against three glycan hole mutants with respect to BG505.T332N. The heat-inactivated rabbit plasma was diluted 100-fold as the starting point and subjected to a 3-fold dilution series in the TZM-bl assay. The %neutralization values obtained from the first dilution are reported in Fig. 3F.
