# Supplementary material for: Neutralizing Antibodies Induced by First-Generation gp41-Stabilized HIV-1 Envelope Trimers and Nanoparticles
Source: mBio. 2021 Jun 22;12(3):e00429-21. doi: 10.1128/mBio.00429-21 (PMC8262854; doi:10.1128/mBio.00429-21)
Supplement: FIG S7 [file mbio.00429-21-sf007.pdf]

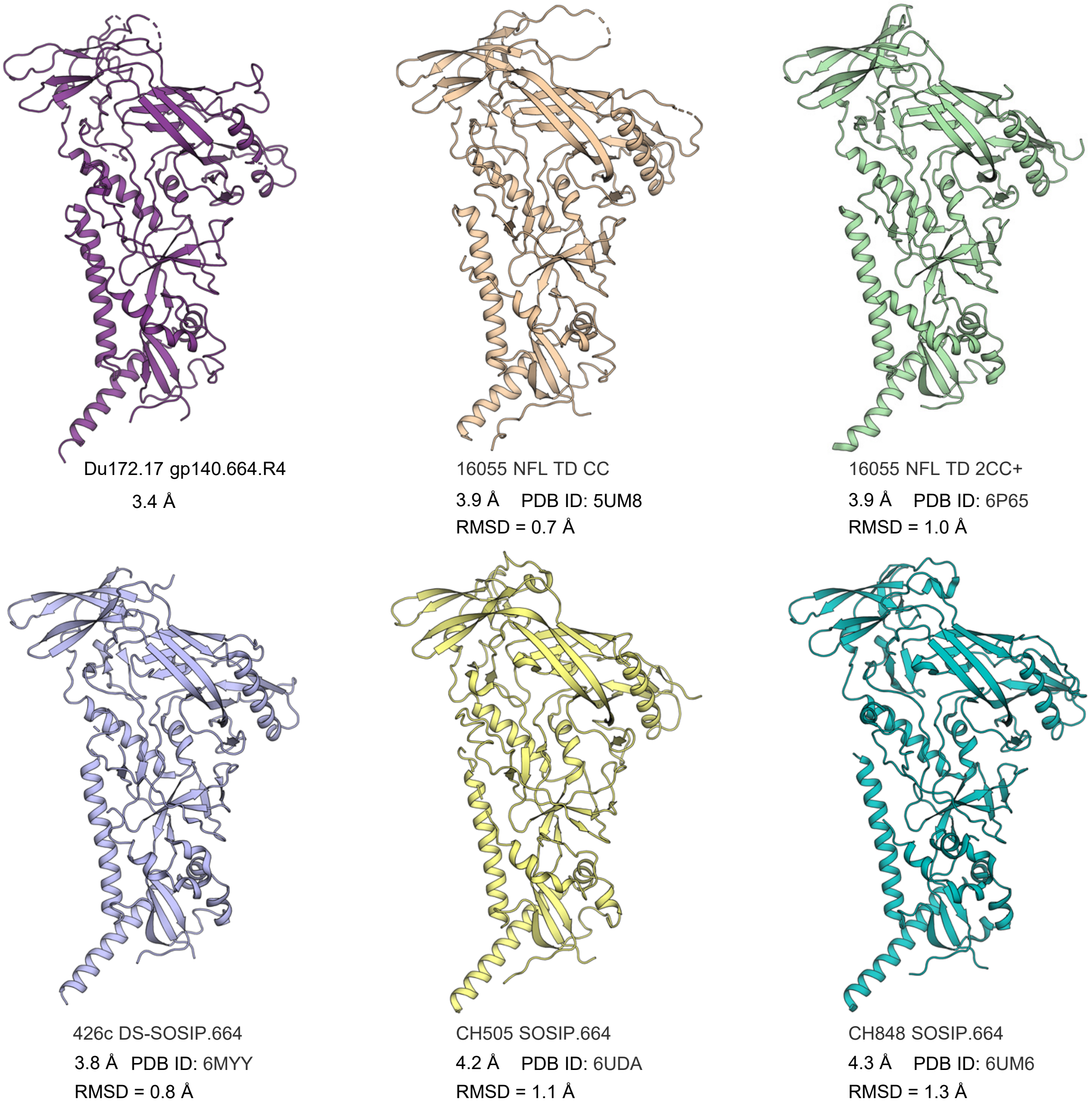

Fig S7 Structural comparison of HIV-1 Envs across clade C isolates. Ribbons view of two crystal structures (Du172.17 here and PDB ID: 5UM8) and four cryo-EM models (PDB IDs: 6P65, 6MYY, 6UDA, and 6UM6) obtained for clade C isolates. The Cα RMSD after superposition of each structure on Du172.17 gp140.664.R4 is also shown.
