# Supplementary material for: Neutralizing Antibodies Induced by First-Generation gp41-Stabilized HIV-1 Envelope Trimers and Nanoparticles
Source: mBio. 2021 Jun 22;12(3):e00429-21. doi: 10.1128/mBio.00429-21 (PMC8262854; doi:10.1128/mBio.00429-21)
Supplement: FIG S8 [file mbio.00429-21-sf008.pdf]

Fig S8

SOSIP.664	N88	N130	N139	N142	N148	N156	N160	N186	N189	N197	N230	N234	N241	N262	N276	N289	N295	N301	N332	N339	N355	N392	N406	N412	N442	N448	N463	N611	N618	N625	N637
High Mannose	95	99	30	0	100	n.d.	99	n.d.	n.d.	76	100	n.d.	n.d.	100	99	n.d.	100	n.d.	100	100	14	100	0	100	100	100	7	0	n.d.	39	91
M9	0	41	0	0	0		10			9	80			82	4		100		90	31	0	0	0	100	52	76	0	0		0	4
M8	16	44	0	0	0		48			28	20			15	70		0		9	51	0	100	0	0	42	20	0	0		2	27
M7	21	9	0	0	100		20			13	0			2	14		0		0	13	1	0	0	0	5	4	0	0		3	33
M6	29	4	5	0	0		10			7	0			1	6		0		0	4	2	0	0	0	1	1	0	0		5	7
M5	20	2	25	0	0		9			16	0			0	3		0		0	0	10	0	0	0	0	0	5	0		24	19
M4	6	0	0	0	0		1			0	0			0	1		0		0	0	0	0	0	0	0	0	0	0		0	0
M3	0	0	0	0	0		0			0	0			0	0		0		0	0	0	0	0	0	0	0	0	0		0	0
Hybrid	3	0	0	0	0		1			2	0			0	0		0		0	0	0	0	0	0	0	0	0	0		3	0
Fhybrid	0	0	0	0	0		0			2	0			0	0		0		0	0	1	0	0	0	0	0	0	0		1	0
A1	1	0	0	0	0		0			1	0			0	0		0		0	0	1	0	0	0	0	0	1	0		4	0
FA1	0	0	11	0	0		0			4	0			0	0		0		0	0	6	0	0	0	0	0	3	0		4	4
A2/A1B	1	0	0	0	0		0			0	0			0	0		0		0	0	0	0	0	0	0	0	6	0		9	0
FA2/FA1B	1	0	12	6	0	n.d.	1	n.d.	n.d.	10	0	n.d.	n.d.	0	0	n.d.	0	n.d.	0	0	16	0	47	0	0	0	21	25	n.d.	23	4
A3/A2B	0	0	0	0	0		0			0	0			0	0		0		0	0	0	0	0	0	0	0	1	5		1	0
FA3/FA2B	1	0	47	56	0		0			7	0			0	0		0		0	0	50	0	53	0	0	0	46	67		20	0
A4/A3B	0	0	0	0	0		0			0	0			0	0		0		0	0	0	0	0	0	0	0	0	0		0	0
FA4/FA3B	0	0	0	38	0		0			1	0			0	0		0		0	0	12	0	0	0	0	0	15	3		0	0
Unoccupied	0	0	0	0	0	0	0	0	0	0	0	0	0	0	0	0	0	0	0	0	1	0	0	0	0	0	0	0	0	0	1

gp140.664.R4	N88	N130	N139	N142	N148	N156	N160	N186	N189	N197	N230	N234	N241	N262	N276	N289	N295	N301	N332	N339	N355	N392	N406	N412	N442	N448	N463	N611	N618	N625	N637
High mannose	99	99	34	0	100	n.d.	98	n.d.	n.d.	96	100	n.d.	n.d.	100	100	n.d.	100	100	100	100	21	100	0	100	100	100	10	67	0	51	99
M9	0	47	0	0	0		38			38	88			87	15		100	86	93	36	0	0	0	100	60	77	0	0	0	0	11
M8	23	45	0	0	0		46			42	12			13	75		0	14	6	49	1	82	0	0	32	20	0	0	0	2	34
M7	24	4	7	0	68		7			8	0			0	8		0	0	0	12	2	18	0	0	7	3	1	5	0	11	31
M6	31	2	5	0	0		4			4	0			0	2		0	0	0	3	3	0	0	0	0	0	0	4	0	8	12
M5	15	1	21	0	32		3			4	0			0	0		0	0	0	0	13	0	0	0	0	0	7	59	0	25	7
M4	4	0	2	0	0		1			0	0			0	0		0	0	0	0	1	0	0	0	0	0	1	0	0	0	0
M3	0	0	0	0	0		0			0	0			0	0		0	0	0	0	0	0	0	0	0	0	1	0	0	0	0
Hybrid	1	0	0	0	0		0			0	0			0	0		0	0	0	0	0	0	0	0	0	0	0	0	0	4	3
Fhybrid	0	0	0	0	0		0			0	0			0	0		0	0	0	0	1	0	0	0	0	0	0	0	0	1	0
A1	1	0	1	0	0		1			0	0			0	0		0	0	0	0	1	0	0	0	0	0	0	2	0	5	0
FA1	0	0	7	0	0		0			0	0			0	0		0	0	0	0	7	0	0	0	0	0	4	4	0	4	0
A2/A1B	0	0	0	0	0		0			0	0			0	0		0	0	0	0	0	0	0	0	0	0	0	0	0	10	0
FA2/FA1B	0	0	14	33	0	n.d.	0	n.d.	n.d.	1	0	n.d.	n.d.	0	0	n.d.	0	0	0	0	18	0	100	0	0	0	25	9	51	19	1
A3/A2B	0	0	0	0	0		0			0	0			0	0		0	0	0	0	0	0	0	0	0	0	0	0	0	1	0
FA3/FA2B	0	0	45	37	0		0			1	0			0	0		0	0	0	0	42	0	0	0	0	0	52	18	49	10	0
A4/A3B	0	0	0	0	0		0			0	0			0	0		0	0	0	0	0	0	0	0	0	0	0	0	0	0	0
FA4/FA3B	0	0	0	30	0		0			0	0			0	0		0	0	0	0	7	0	0	0	0	0	8	0	0	0	0
Unoccupied	0	0	0	0	0	0	0	0	0	1	0	0	0	0	0	0	0	0	0	0	3	0	0	0	0	0	0	0	0	0	0

UFO.664	N88	N130	N139	N142	N148	N156	N160	N186	N189	N197	N230	N234	N241	N262	N276	N289	N295	N301	N332	N339	N355	N392	N406	N412	N442	N448	N463	N611	N618	N625	N637
High mannose	88	100	23	0	100	100	100	n.d	n.d.	99	100	100	100	100	100	n.d.	100	100	100	100	11	100	0	100	100	100	4	24	0	0	84
M9	0	70	0	0	0	100	60			58	83	70	59	89	20		100	71	94	42	0	28	0	100	78	75	0	0	0	0	0
M8	8	22	0	0	0	0	35			33	17	30	41	10	73		0	29	5	46	0	44	0	0	19	22	0	0	0	0	4
M7	12	4	0	0	25	0	5			4	0	0	0	1	6		0	0	1	10	0	29	0	0	2	3	0	0	0	0	38
M6	21	1	0	0	0	0	0			2	0	0	0	0	1		0	0	0	2	0	0	0	0	0	1	0	0	0	0	17
M5	31	2	23	0	75	0	0			2	0	0	0	0	0		0	0	0	0	8	0	0	0	0	0	3	20	0	0	16
M4	7	0	0	0	0	0	0			0	0	0	0	0	0		0	0	0	0	0	0	0	0	0	0	0	0	0	0	1
M3	1	0	0	0	0	0	0			0	0	0	0	0	0		0	0	0	0	0	0	0	0	0	0	0	0	0	0	0
Hybrid	7	0	0	0	0	0	0			0	0	0	0	0	0		0	0	0	0	0	0	0	0	0	0	0	2	0	0	4
Fhybrid	1	0	0	0	0	0	0			0	0	0	0	0	0		0	0	0	0	1	0	0	0	0	0	0	1	0	0	3
A1	5	0	0	0	0	0	0			0	0	0	0	0	0		0	0	0	0	1	0	0	0	0	0	2	2	0	0	1
FA1	0	0	12	0	0	0	0			0	0	0	0	0	0		0	0	0	0	6	0	0	0	0	0	5	6	1	0	5
A2/A1B	5	0	0	0	0	0	0			0	0	0	0	0	0		0	0	0	0	0	0	0	0	0	0	8	0	0	0	0
FA2/FA1B	1	0	17	40	0	0	0	n.d	n.d.	0	0	0	0	0	0	n .d.	0	0	0	0	19	0	41	0	0	0	26	11	34	9	8
A3/A2B	1	0	0	0	0	0	0			0	0	0	0	0	0		0	0	0	0	0	0	0	0	0	0	1	0	0	0	0
FA3/FA2B	1	0	49	44	0	0	0			0	0	0	0	0	0		0	0	0	0	54	0	58	0	0	0	45	43	64	79	0
A4/A3B	0	0	0	0	0	0	0			0	0	0	0	0	0		0	0	0	0	0	0	0	0	0	0	0	0	0	0	0
FA4/FA3B	0	0	0	16	0	0	0			0	0	0	0	0	0		0	0	0	0	9	0	0	0	0	0	8	4	0	12	0
Unoccupied	0	0	0	0	0	0	0	0	0	1	0	0	0	0	0	0	0	0	0	0	0	0	0	0	0	0	0	9	0	0	2

Fig S8 Site-specific *N*-linked glycan analysis of Du172.17 SOSIP.664, HR1-redesigned (gp140.664.R4), and UFO.664 trimers produced in HEK293F cells. Quantification of site-specific glycan occupancy and composition. The table shows the compositions found at each site. Compositions corresponding to oligomannose/hybrid-type glycans are colored in green and fully processed complex type glycans are colored in magenta. The proportion of peptides at each lacking an attached glycan are colored in grey. Oligomannose-type glycans are categorized according to the number of mannose residues, hybrid-type glycans according to the presence/absence of fucose, and complex-type glycans according to the number of processed antenna and the presence/absence of fucose.
