# Supplementary material for: Neutralizing Antibodies Induced by First-Generation gp41-Stabilized HIV-1 Envelope Trimers and Nanoparticles
Source: mBio. 2021 Jun 22;12(3):e00429-21. doi: 10.1128/mBio.00429-21 (PMC8262854; doi:10.1128/mBio.00429-21)
Supplement: FIG S9 [file mbio.00429-21-sf009.pdf]

Fig S9

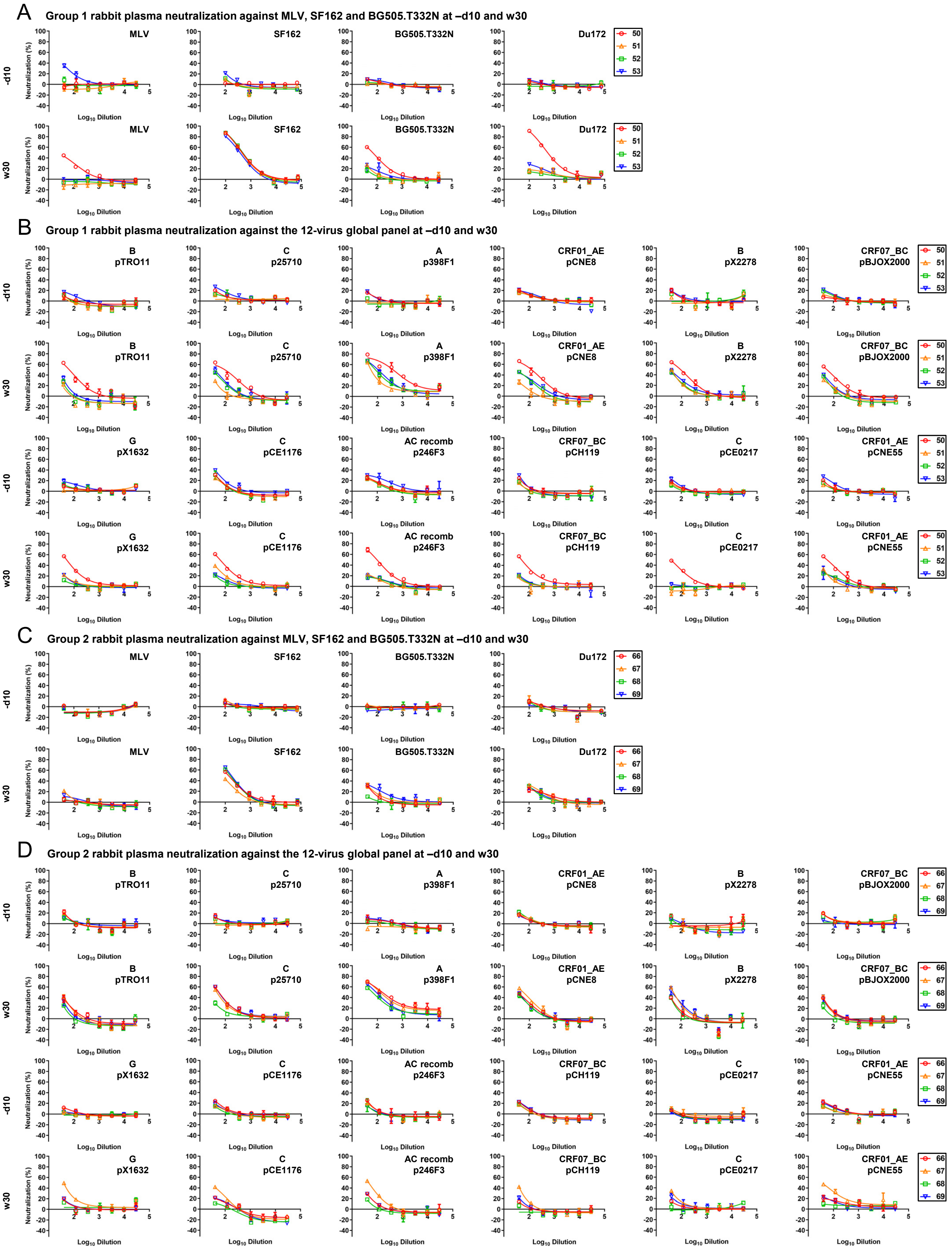
A
Group 1 rabbit plasma neutralization against MLV, SF162 and BG505.T332N at –d10 and w30
-d10
w30
Neutralization (%)
Log10 Dilution
MLV
SF162
BG505.T332N
Du172
50
51
52
53
B
Group 1 rabbit plasma neutralization against the 12-virus global panel at –d10 and w30
B pTRO11
C p25710
A p398F1
CRF01_AE pCNE8
B pX2278
CRF07_BC pBJOX2000
G pX1632
C pCE1176
AC recomb p246F3
CRF07_BC pCH119
C pCE0217
CRF01_AE pCNE55
C
Group 2 rabbit plasma neutralization against MLV, SF162 and BG505.T332N at –d10 and w30
66
67
68
69
D
Group 2 rabbit plasma neutralization against the 12-virus global panel at –d10 and w30

Fig S9

E Group 3 rabbit plasma neutralization against MLV, SF162 and BG505.T332N at –d10 and w30

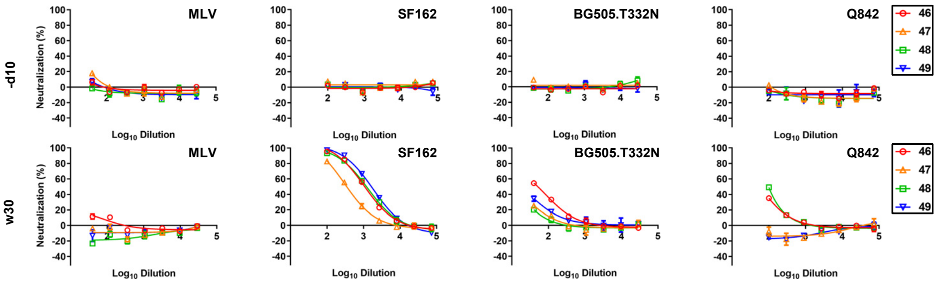

F Group 3 rabbit plasma neutralization against the 12-virus global panel at –d10 and w30

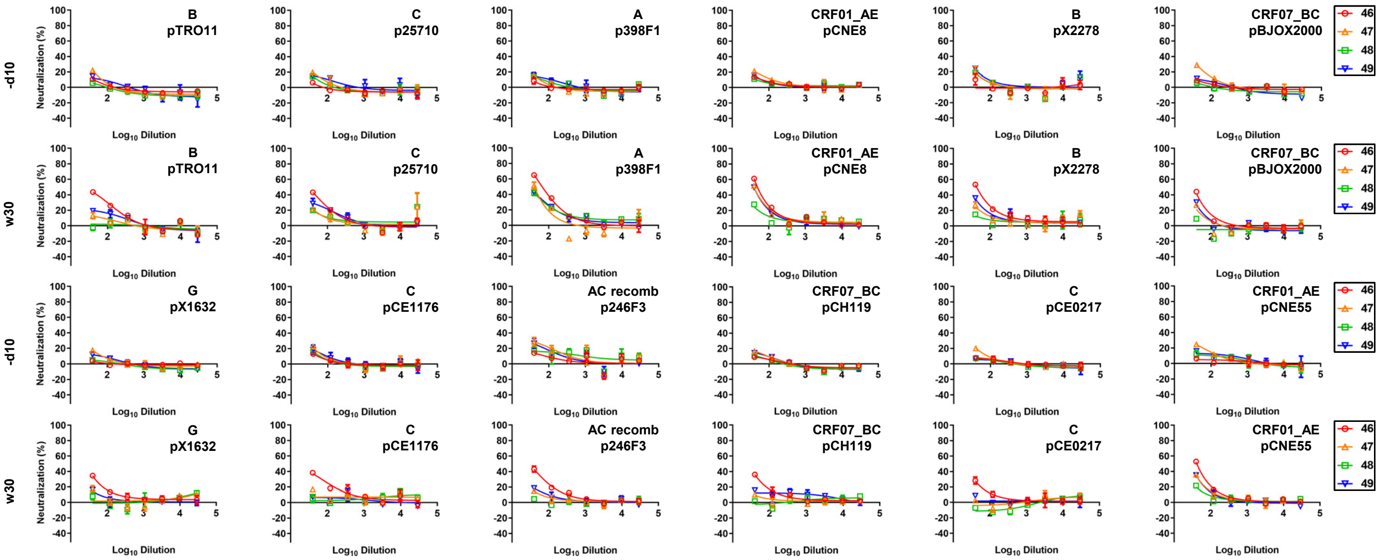

G Group 1 rabbit plasma neutralization against at 6 time points

Group 2 rabbit plasma neutralization against at 6 time points

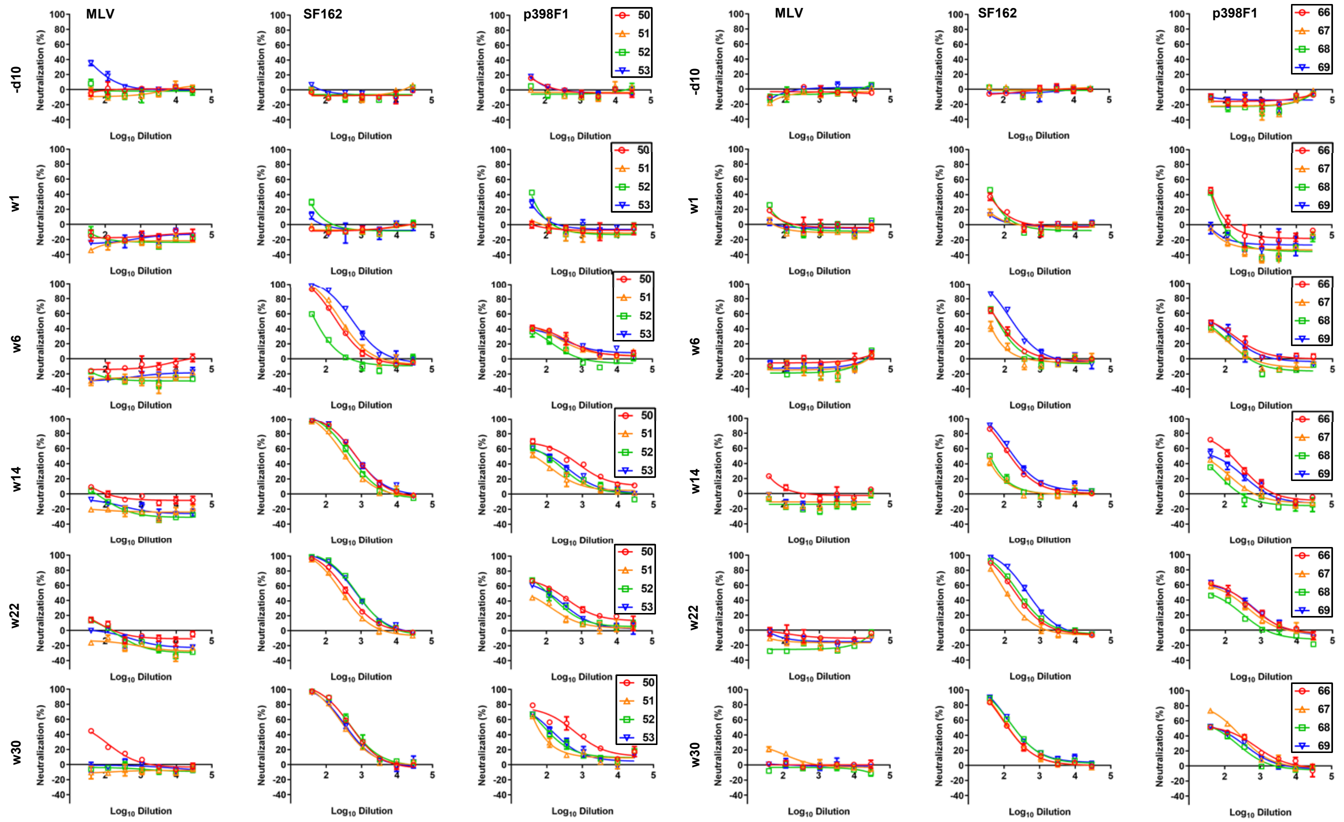

Fig S9

Fig S9 Rabbit plasma neutralization from three non-BG505 Env-immunized rabbit groups. Three groups of rabbits were immunized with Du172.17 UFO-BG trimer, Du172.17 gp140.664.R4-FR nanoparticle, and Q842-d12 UFO-BG trimer. (**A**) Neutralization of MLV, tier 1 clade B SF162, tier 2 clade A BG505.T332N, and tier 2 clade C Du172.17 by day -10 (-d10) and week 30 (w30) rabbit plasma from the Du172.17 trimer group. (**B**) Neutralization of all 12 isolates from a global panel by day -10 (-d10) and week 30 (w30) rabbit plasma from the Du172.17 trimer group. (**C**) Neutralization of MLV, SF162, BG505.T332N, and Du172.17 by day -10 (-d10) and week 30 (w30) rabbit plasma from the Du172.17 ferritin nanoparticle group. (**D**) Neutralization of all 12 isolates from a global panel by day -10 (-d10) and week 30 (w30) rabbit plasma from the Du172.17 ferritin nanoparticle group. (**E**) Neutralization of MLV, SF162, BG505.T332N, and tier 2 clade A Q842-d12 by day -10 (-d10) and week 30 (w30) rabbit plasma from the Q842-d12 trimer group. (**F**) Neutralization of all 12 isolates from a global panel by day -10 (-d10) and week 30 (w30) rabbit plasma from the Q842-d12 trimer group. The heat-inactivated plasma was diluted 100-fold for autologous virus and tier 1 SF162 and subjected to a 3-fold dilution series in the TZM-bl assay. To increase the sensitivity of detection, heat-inactivated plasma was diluted 40-fold for MLV and all other heterologous tier 2 isolates and followed by a 3-fold dilution series in the TZM-bl assay. ID_{50} titers for plots in (A) – (F) are summarized in FIG 4D. (**G**) Longitudinal rabbit plasma neutralization from two Du172.17 rabbit groups**.** Left: Neutralization of MLV, tier 1 clade B SF162, and tier 2 clade A p398F1 by day -10 (-d10) and weeks 1, 6, 14, 22, and 30 rabbit plasma from the Du172.17 trimer group. Right**:** Neutralization of MLV, tier 1 clade B SF162, and tier 2 clade A p398F1 by day -10 (-d10) and weeks 1, 6, 14, 22, and 30 rabbit plasma from the Du172.17 gp140.664.R4-FR nanoparticle group. In this analysis, the heat-inactivated plasma was diluted 40-fold for both SF162 and p398F1 and then subjected to a 3-fold dilution series in the TZM-bl assay. ID_{50} titers for plots in (G) are shown in Fig. 4E.
