# Supplementary material for: Neutralizing Antibodies Induced by First-Generation gp41-Stabilized HIV-1 Envelope Trimers and Nanoparticles
Source: mBio. 2021 Jun 22;12(3):e00429-21. doi: 10.1128/mBio.00429-21 (PMC8262854; doi:10.1128/mBio.00429-21)
Supplement: TABLE S1 [file mbio.00429-21-st001.docx]

| **Table S1.** **X-ray crystallographic data collection and refinement statistics.** | | | | |
| --- | --- | --- | --- | --- |
| **Data collection** | BG505 gp120 core, Fabs M4H2K1, 17b | Fab M4H2K1 | Du172.17 gp140.664.R4, Fabs PGT124, 35O22 |  |
| X-ray Source | APS 23ID-B | APS 23ID-D | APS 23ID-D |  |
| Wavelength (Å) | 1.033 | 1.033 | 1.033 |  |
| Detector | Eiger | Pilatus | Pilatus |  |
| Space group | P2_1_2_1_2 | P3_1_21 | P6_3_ |  |
| Unit cell parameters | a = 204.0, b = 60.6, c = 166.7 Å | a = b = 68.3, c = 184.7 Å | a = b = 127.0, c = 316.5 Å |  |
| Resolution (Å) | 50.00 – 4.30 (4.73 – 4.63) (4.63 – 4.54)  (4.54 – 4.45) (4.45 – 4.37) (4.37 – 4.30) | 50.00 – 1.50 (1.53 – 1.50)^a^ | 50.00 – 3.40 (3.46 – 3.40)^a^ |  |
| Observations | 109,015 | 929,037 | 248,831 |  |
| Unique reflections | 12,843 (280) ^a^ | 81,206 (3,998) ^a^ | 38,788 (1614) ^a^ |  |
| Redundancy | 8.5 (1.8) ^a^ | 11.4 (11.1) ^a^ | 6.4 (3.7) ^a^ |  |
| Completeness (%) | 86.8 (80.4) (67.2) (61.8) (52.6) (45.6) (39.4) | 99.8 (99.5) ^a^ | 97.6 (81.1) |  |
| <*I/σ_I_*>^b^ | 13.0 (1.0) ^a^ | 34.9 (2.0) ^a^ | 8.6 (1.0) ^a^ |  |
| *R*_sym_ ^c^  *R_pim_* ^c^  CC_1/2_ | 0.21 (0.83) ^a^  0.06 (0.50) ^a^  0.86 (0.39)^a^ | 0.08 (0.99) ^a^  0.02 (0.29) ^a^  0.94 (0.73)^a^ | 0.21 (1.00) ^a^  0.08 (0.48) ^a^  0.85 (0.45)^a^ |  |
| **Refinement statistics** |  |  |  |  |
| Resolution (Å) | 43.04 – 4.30 | 49.88 – 1.50 | 49.53 – 3.40 |  |
| Reflections (work) | 12,392 | 81,150 | 38,200 |  |
| *R*_cryst_ (%) ^d^ / *R*_free_ (%) ^e^ | 30.1 / 33.3 | 18.3 / 21.6 | 24.2 / 29.17 |  |
| No. atoms  Protein / Ligands  Glycan  Water | 9429  282  - | 3347 / 17  -  624 | 11322  726  - |  |
| Average *B*-value (Å^2^)  Protein  Glycan  Water | 172  89  - | 24  -  36 | 108  147  - |  |
| Wilson *B*-value (Å^2^) | 139 | 18 | 93 |  |
| RMSD from ideal geometry | |  |  |  |
| Bond length (Å) | 0.004 | 0.009 | 0.002 |  |
| Bond angles (°) | 0.85 | 1.16 | 0.69 |  |
| Ramachandran statistics (%) ^f^ | |  |  |  |
| Favored | 95.05 | 97.71 | 91.39 |  |
| Allowed | 4.37 | 2.29 | 7.65 |  |
| Outliers | 0.58 | 0 | 0.96 |  |
| PDB ID | 7KLC | 7KKZ | 7KMD |  |
| ^a^ Numbers in parentheses refer to the highest resolution shell.  ^b^ Calculated as average(*I*)/average(σ*I*)  ^c^*R*_sym_ = Σ*_hkl_*Σ*_i_* \| *I_hkl,i_* - <*I_hkl_*> \| / Σ*_hkl_*Σ*_i_I_hkl,I_*, where *I_hkl,i_* is the scaled intensity of the *i*^th^ measurement of reflection h, k, l, <*I_hkl_*> is the average intensity for that reflection, and *n* is the redundancy. *R_pim_* is a redundancy-independent measure of the quality of intensity measurements. *R_pim_* = Σ*_hkl_* (1/(*n*-1))^1/2^ Σ*_i_* \| *I_hkl,i_* - <*I_hkl_*> \| / Σ*_hkl_* Σ*_i_ I_hkl,I_*, where *I_hkl,i_* is the scaled intensity of the *i*^th^ measurement of reflection h, k, l, < *I_hkl_* > is the average intensity for that reflection, and *n* is the redundancy.  ^d^*R*_cryst_ = Σ*_hkl_* \| *F*_o_ - *F*_c_ \| / Σ*_hkl_* \| *F*_o_ \| × 100  ^e^*R*_free_ was calculated as for *R*_cryst_, but on a test set comprising 5% of the data excluded from refinement.  ^f^These values were calculated using MolProbity (<http://molprobity.biochem.duke.edu/>). | | | | |
